# Harnessing Lessons from Gel-Based and Advanced Biomaterial Therapeutics to Enable Direct Cellular Reprogramming

**DOI:** 10.3390/gels12060486

**Published:** 2026-06-01

**Authors:** Daniel González-Nieto, José Pérez-Rigueiro, Francisco J. Rojo, Fivos Panetsos, Gustavo V. Guinea

**Affiliations:** 1 Center for Biomedical Technology, Universidad Politécnica de Madrid, 28223 Pozuelo de Alarcón, Spain; jose.perez@ctb.upm.es (J.P.-R.); fj.rojo@upm.es (F.J.R.); gustavovictor.guinea@ctb.upm.es (G.V.G.); 2 Departamento de Tecnología Fotónica y Bioingeniería, ETSI Telecomunicaciones, Universidad Politécnica de Madrid, 28040 Madrid, Spain; 3 Centro de Investigación Biomédica en Red de Bioingeniería, Biomateriales y Nanomedicina (CIBER–BBN), Instituto de Salud Carlos III, 28029 Madrid, Spain; 4 Departamento de Ciencia de Materiales, ETSI Caminos, Canales y Puertos, Universidad Politécnica de Madrid, 28040 Madrid, Spain; 5 Biomaterials and Regenerative Medicine Group, Instituto de Investigación Sanitaria del Hospital Clínico San Carlos (IdISSC), 28040 Madrid, Spain; 6 Neural Plasticity Research Group, Instituto de Investigación Sanitaria del Hospital Clínico San Carlos (IdISSC), 28040 Madrid, Spain; fivos@ucm.es; 7 Neurocomputing and Neurorobotics Research Group, Universidad Complutense de Madrid, 28040 Madrid, Spain

**Keywords:** biomaterials, direct cell reprogramming, tissue engineering, brain, heart

## Abstract

Direct cellular reprogramming, the conversion of one somatic cell type into another, represents a remarkable advancement in regenerative medicine. Its potential to transform fibrotic tissue into functional parenchyma underscores its therapeutic promise. However, several critical challenges remain unresolved, including limited reprogramming efficiency, the long-term functional stability of converted cells, their integration within pre-existing cellular circuits, and safety concerns related to transgene integration and immunological responses to reprogramming-based viral vectors. Approaches based on the exogenous administration of recombinant proteins and miRNAs have also emerged, though these rely on factors that are naturally prone to exhaustion and degradation, potentially restricting their efficacy. This review is divided into three main sections. The first part addresses direct cellular reprogramming in the context of other cell-based applications, outlining its main applications and current biological limitations. The second part examines how different biomaterials, ranging from hydrogel scaffolds to nanoparticles, can modulate direct cellular reprogramming by providing mechanical and topographical cues and by enabling tighter control over the concentration and spatiotemporal dynamics of reprogramming factors and viral vectors. The third part discusses key findings in biomaterial-assisted reprogramming strategies, highlighting emerging opportunities for clinically translatable approaches. The convergence of regenerative biology and biomaterials science may ultimately generate advanced gel-based and hybrid cellular reprogramming platforms for in vitro testing and, in situ applications, for promoting cell fate stabilization and facilitating the regeneration of damaged tissues and organs.

## 1. Introduction

Mammals, including humans, exhibit limited regenerative capacity in most tissues and organs. This limited ability contrasts sharply with the remarkable regenerative potential of other species such as the axolotl, an amphibian capable of fully regenerating a limb after amputation [1]. The inherent regenerative capacity of the axolotl has long served, in some way, as a major source of inspiration in the search for strategies capable of promoting tissue repair and regeneration in humans. In recent decades, the field of regenerative medicine has undergone a profound and rapid advancement. This progress has been driven first by the characterization and use of somatic and stem cells, later by the development of induced pluripotent stem cells, and more recently by the emergence of direct cellular reprogramming technologies.

Direct cellular reprogramming (DCR) likely represents the latest technological milestone for the mimetic recreation of tissues and organs, both for diagnostics and transplantation, as well as for the in situ replacement of damaged tissues. However, this technology faces several bottlenecks, including low reprogramming efficiency both in vitro and in vivo, limited stability of reprogramming factors, heterogeneity of the target cell populations, reliance on viral vectors with associated risks such as insertional mutagenesis and potential immortalization, and immune responses even to non-integrating viral vectors such as adenoviruses. The use of natural and synthetic materials in biomedical applications is no longer restricted to the preclinical context, as an increasing number of applications using relatively simple biomaterial formats have reached clinical practice, for example, as structural supports, drug delivery systems, cell grafts, or diagnostic procedures. Nevertheless, there is still significant room for growth.

Although the principles of DCR date back to the 1980s, when the conversion of fibroblasts into muscle cells was achieved via the introduction of the MyoD factor [2], its full potential only began to be exploited decades later, following key advances in cellular reprogramming. Since then, a wide variety of biomaterials and formulations have been employed to overcome the primary limitations of DCR.

While earlier reviews have addressed either the biological mechanisms of reprogramming [3] or the design and fabrication of biomaterials to address the main challenges in biomedicine and health [4,5,6,7], only a limited number of studies have specifically addressed their intersection in the context of direct reprogramming [8,9]. These works have provided valuable foundational insights into how biomaterials can influence cell fate. However, a comprehensive and up-to-date analysis integrating biomaterial design, fabrication strategies, and their functional impact on direct reprogramming remains lacking. In this review, we aim to bridge this gap by providing an integrative perspective on how advanced biomaterial systems, including hydrogels, nanomaterials, and engineered substrates, can be rationally designed to enhance in situ direct cellular reprogramming. In particular, we focus on how these platforms can help overcome key biological and translational bottlenecks in cardiac and neural regeneration. The manuscript is structured into three main sections. The first part establishes the clinical context by reviewing the evolution of stem cell-based therapies and their limitations in non-hematopoietic tissues. This section introduces the roadmap of DCR, including its main applications and a detailed analysis of the biological bottlenecks that currently hinder its success. The second part delves into the physicochemical and design principles of biomaterials relevant to reprogramming. The third part discusses key findings in biomaterial-assisted reprogramming strategies, highlighting emerging opportunities for clinically translatable approaches. Finally, at the end of this review we include an outlook section discussing the current clinical challenges in the field of direct cellular reprogramming and how biomaterials may help address these challenges to facilitate translation to patients.

## 2. Rethinking Stem Cell-Based Therapies: Conceptual Limits and the Shift Toward Direct Reprogramming

### 2.1. Limited Regenerative Efficacy of Stem Cells in Non-Hematopoietic Tissues

For decades, the use of stem cells has reflected the scientific and medical community’s interest in protecting and repairing damaged tissues. However, few stem cell-based applications have proven clinically useful in actual patient treatments. One of the few successful applications is bone marrow transplantation, a procedure that has convincingly demonstrated the ability of stem cells to self-renew and fully regenerate the hematopoietic system of a patient. This procedure involves the infusion of hematopoietic stem and progenitor cells from a donor into a recipient patient to reconstitute the host bone marrow after irradiation, chemotherapy, or a combination of both, a procedure first performed nearly 70 years ago [10]. It has also been demonstrated that a single hematopoietic stem cell is theoretically sufficient to sustain long-term hematopoietic system reconstitution [11,12]. Although not yet routinely applied, initial steps have been taken toward transplantation of genetically corrected hematopoietic stem cells, for example, using clustered regularly interspaced short palindromic repeats (CRISPR) or second-generation Cas9 editors. In this context, clinical trials have already been conducted for the treatment of β-hemoglobinopathies, such as sickle cell disease, and other conditions like cerebral adrenoleukodystrophy, although safe and efficient protocols for introducing precise genomic changes are still required [13,14].

In contrast to the field of hematology, the use of stem cells for regeneration of non-hematopoietic tissues in patients remains limited. Although clinical trials have explored stem cell-based therapies in cardiac and neural tissues, results have been mixed: certain studies show encouraging effects, whereas others remain inconclusive or lack of positive outcomes [15,16,17,18,19,20]. Evidence of this is that, today, stem cell-based therapies for brain or cardiac injury are not yet part of routine clinical practice. The clinical benefits of bone marrow transplants likely lie in the specific nature of the problem and the unique properties of hematopoietic tissue compared to other tissues. A defining feature of hematopoietic tissue is its capacity to continuously produce and replace blood cells, even during early gestation [21]. This feature represents a significant difference from other, more permanent tissues such as the brain or the heart, where the generation of new functional cells is virtually nonexistent.

Although long debated, studies have not demonstrated that cardiac stem cells generate new functional cardiomyocytes after cardiac injury, at least in mammals [22]. The fibrotic response that occurs following cardiac injury is not regenerative, but rather protective, preventing the rupture of healthy cardiac tissue adjacent to the lesion through the formation of a fibrotic scar. It is, however, remarkable that in other vertebrate species, spontaneous signs of cardiac regeneration have been observed. For example, the zebrafish heart can partially regenerate following mild ventricular damage. This process occurs through the endogenous reprogramming of preexisting adult cardiomyocytes, a mechanism that has, in part, inspired current direct reprogramming technologies. Dedifferentiation of these cardiomyocytes is driven by the silencing of tissue-specific genes and the induction of embryonic cardiac genes, leading to a stage of controlled proliferation, followed by upregulation of tissue-specific genes that guide differentiation and the formation of new cardiac tissue [23,24]. Initially, it was demonstrated that factors such as PLK1 and MPS1 induce changes in preexisting cardiomyocytes adjacent to the injury, including disorganization of actin and myosin filaments, sarcomere disruption, and reentry into the cell cycle [25]. Subsequently, a variety of factors have been identified that may contribute to this regenerative process. Among them, the anti-fibrotic factor BMP7, which not only participates in cardiac regeneration in zebrafish, but whose chronic administration also appears to stimulate regenerative capacity in mice following heart injury [26]. Another studied factor is HMGA1, which similarly promotes cardiomyocyte proliferation and regeneration in both zebrafish and mice [27].

Regarding the central nervous system, there remains controversy about the presence of functional stem cells in the adult human brain capable of continuously producing neurons. This debate, fueled by the use of different technologies and protocols to detect neural stem and progenitor cells, has focused primarily on the human hippocampus, with a trend over the past five years supporting adult hippocampal neurogenesis [28,29,30,31]. However, the physiological role of neurogenesis in the hippocampus, other subcortical regions, or the cerebral cortex remains to be defined. A key distinction compared to the hematopoietic system, which can continuously replace blood cells under physiological or pathological (i.e., sublethal irradiation or chemotherapy) conditions [32], is that adult neurogenesis does not appear to contribute to the replacement or regeneration of damaged brain tissue following injury. When recovery does occur, functional improvement (typically partial) in humans after brain injury is usually mediated by the formation of new compensatory neural circuits from preexisting brain tissue [33], and there is no clear evidence of true regeneration in the human brain.

### 2.2. Challenges in Cell Mobilization and Homing

Different types of stem cells, including hematopoietic and mesenchymal stem cells, as well as embryonic and induced pluripotent stem cells, along with differentiated cells, have been employed in various cell therapy modalities [34]. Some of these strategies have relied on the intravenous infusion of mesenchymal stem cells and hematopoietic stem and progenitor cells [35,36]. Under physiological conditions, hematopoietic stem and progenitor cells (HSC/P) continuously circulate, mobilizing into the bloodstream and subsequently homing back to the bone marrow, maintaining tissue homeostasis [37]. This distinctive migratory property, which is clinically relevant in bone marrow transplantation, appears to be exceptional and likely restricted to hematopoietic and blood compartments. The ability of HSC/P to migrate to distant non-hematopoietic tissues, such as the brain, is exceedingly rare and not well characterized. In contrast, mesenchymal stem cells (MSCs) are normally scarce in peripheral blood, but their numbers increase markedly following tissue injuries such as trauma, cardiac dysfunction, liver damage, or cancer. Current challenges include accurately detecting these cells and characterizing their functional, phenotypic, and molecular properties [38,39].

Therefore, there is no definitive evidence supporting the mobilization of endogenous stem cells from the bone marrow to injured central nervous system or cardiac tissue. Most reports support endogenous mobilization based on studies involving the infusion of exogenous stem cells [38,40]. Surprisingly, outside the hematopoietic context, many cell therapies assume that exogenously infused stem cells will home to sites of tissue damage. However, some evidence indicates that ex vivo manipulation, such as prolonged culture or expansion, can substantially impair both the migratory capacity and therapeutic efficacy of these cells [41]. By contrast, no mobilization of neural stem cells from the brain to other organs has been reported. However, peripheral neural stem cells (pNSCs) have recently been identified; these cells share multipotent properties and exhibit high transcriptomic and functional similarity with neural stem cells (NSCs), with both populations originating from the neural tube during embryonic development [42].

In the context of the heart, considerable controversy remains. Although G-CSF, a disruptor of the SDF-1α/CXCR4 axis in the bone marrow, successfully mobilized bone marrow-derived adult stem cells (hematopoietic and mesenchymal) into the bloodstream in the STEMMI clinical trial, no clinical benefit was observed in patients with acute myocardial infarction [18]. Although the infused cells were found in blood, there was no evidence of endogenous stem cell mobilization toward the heart [43]. Similarly, another clinical study reported no improvement in patients following intracoronary implantation of autologous bone marrow MSCs [44]. These clinical trials suggest that the endogenous mobilization of stem cells to cardiac tissue is almost negligible. In contrast, alternative studies in rats indicate that endogenous MSCs and hematopoietic progenitors can migrate to infarcted cardiac tissue, with peaks in mobilization observed 1 and 5 days post-infarction for MSCs and hematopoietic progenitors, respectively [45]. In another study in domestic pigs, intravenous infusion of bone marrow mononuclear cells was ineffective in targeting the infarcted myocardium [46].

Recently, pluripotent stem cells have been implanted in cardiac tissue in non-human primates, resulting in functional improvement [47]. In another study in mice, the implantation of neural stem cells into perilesional brain tissue following a cerebral infarction allowed the cells to functionally integrate into the host circuitry, responding to peripheral sensory stimuli in the previously damaged somatosensory cortex [48]. The lack of spontaneous regeneration in damaged cardiac and brain tissue in adult humans aligns with the limited clinical efficacy observed in cell-based therapies, yet this stands in stark contrast to animal models, which consistently demonstrate the potential of such therapies to restore the function of damaged tissues. Although certain stem cell therapies have demonstrated clinical utility in other settings, such as hematopoietic stem cell transplantation or the use of MSCs in immune-mediated disorders like Crohn’s disease, these successes have not translated to tissues with limited intrinsic regenerative capacity. This apparent refractoriness of human permanent tissues may reflect the inability of exogenously delivered stem cells, when placed into the adult human microenvironment, to mimic endogenous regenerative behaviors that are themselves minimal, or virtually absent, in the heart and brain.

### 2.3. Paracrine Signaling vs. True Regeneration

Although stem cells have limited capacity to directly replace damaged tissue in largely non-regenerative organs such as the heart or the brain, numerous studies instead emphasize their role in modulating the injured tissue microenvironment. A growing body of evidence supports the immunomodulatory, antioxidant, and cytoprotective effects of stem cells, including the provision of survival signals in cardiac and nervous tissues [49,50]. These actions are mediated through paracrine mechanisms, either by the release of soluble factors (the secretome) or exosomes carrying bioactive molecules [49]. Other capacities of stem cells include the secretion of angiogenic factors or chemoattractant molecules that stimulate the recruitment of progenitor cells toward the injury site, at least in animal models.

### 2.4. Reparative-to-Regenerative Transformation: Inducing Functional Competence in Non-Specialized Cells

One of the most promising current strategies in tissue regeneration aims to induce functional changes in situ within resident cells, either by mimicking regenerative processes observed in adult cardiac tissue in some animal species, or by attempting to redirect the injury response from a primarily reparative to a regenerative program.

In 1962, Dr. John B. Gurdon demonstrated that the nucleus of a differentiated epithelial cell could generate an entire organism, a tadpole, when transplanted into an enucleated egg cell [51]. This early study demonstrated that differentiated cells can revert to less specialized states with multi- or pluripotent potential. Building on this principle, the pioneering work of Takahashi and Yamanaka further established and mechanistically defined cellular reprogramming by identifying a set of transcription factors capable of inducing pluripotency in virtually any somatic cell type [52]. Contrary to the original belief that cell fate is irreversible, Yamanaka’s group developed a technology capable of converting a somatic cell into a pluripotent stem cell (iPSC) through the transduction of four transcription factors (Oct3/4, Sox2, c-Myc, and Klf4). This study established a new paradigm, which over the past two decades has sparked intense research efforts aimed at identifying additional factors and developing more efficient reprogramming protocols. In addition to viral cDNA transduction, cells have been reprogrammed using synthetic mRNAs, gene silencing approaches [53], recombinant proteins [54], or small molecules [55]. Nevertheless, the conversion of somatic cells into pluripotent stem cells for tissue regeneration faces significant challenges that limit its clinical applicability. These include the teratogenic potential of pluripotent stem cells when implanted or generated in vivo, the negative impact of the immune response, primarily mediated by natural killer cells on the efficiency of in vivo reprogramming [56], and potential long-term side effects such as hepatic and intestinal toxicity or premature death [57].

Another transformative approach in regenerative medicine, aligned with the clinical goals of patient safety and therapeutic efficacy, is DCR, which enables the conversion of one somatic cell type into another. DCR can be performed ex vivo or in situ, and can circumvent some of the risks associated with cell transplantation, such as allogeneic rejection, while enabling the generation of functionally reprogrammed cells for tissue repair [53]. Although the foundations of DCR were established nearly 40 years ago [2], the true potential of this technology, much like others such as CRISPR, has been extensively explored over the last two decades. Using DCR, it has been possible to generate a variety of functional cell types both in vitro and in vivo, including neurons [58,59], cardiomyocytes [60,61,62], and even hematopoietic cells [63], from human or mouse fibroblasts. Since the vast majority of human tissues repair through scar formation, cellular reprogramming has been employed to transform this reparative process into a regenerative one. For example, the in vivo conversion of astrocytes into neural progenitors has been achieved through the reintroduction of the transcription factor DLX2 [64]. In another example, cardiac fibroblasts have been converted into functional cardiomyocytes through the expression of three transcription factors: Gata4, Mef2c, and Tbx5 (GMT), illustrating the potential of direct reprogramming to transform scar-forming cells into functional tissue [62].

#### Unresolved Bottlenecks in Direct Cellular Reprogramming

**Figure 1:**
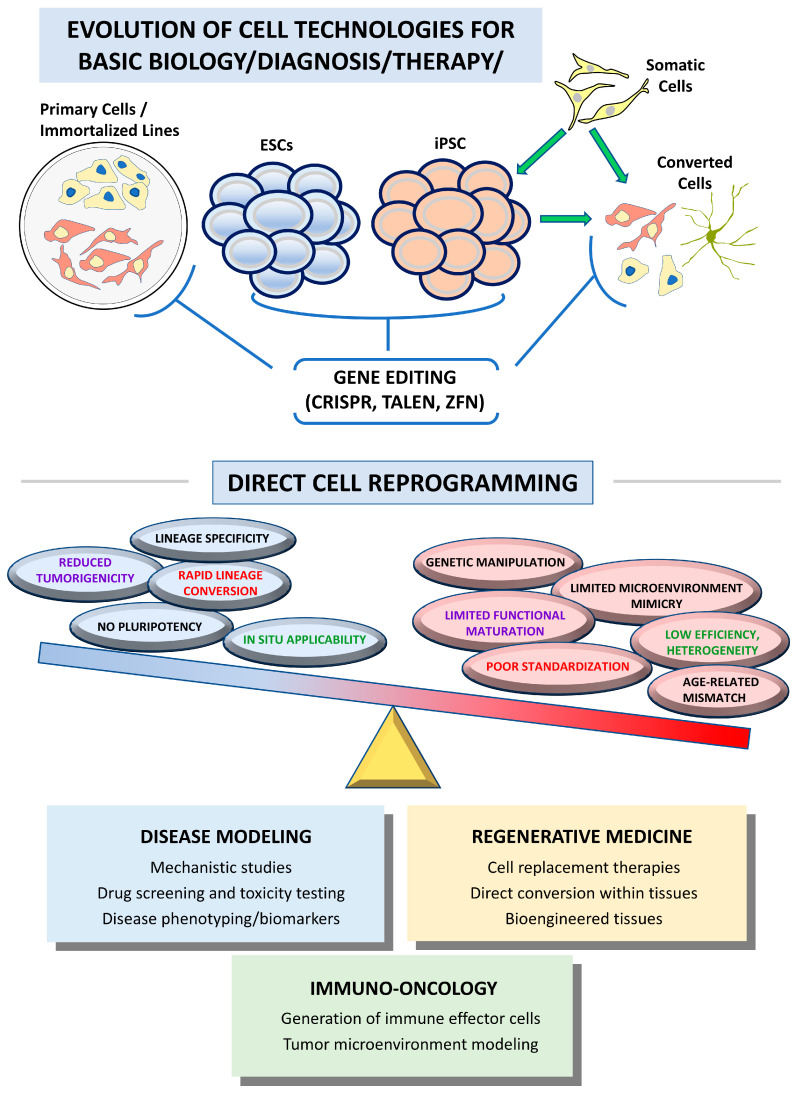
Evolution of Cell-Based Therapies and Current Challenges in Direct Cellular Reprogramming. Top, schematic representation illustrating the evolution of cell-based technologies over the last decades in the field of regenerative medicine. Different strategies have been explored, including transplantation of stem cells, progenitors, precursors, terminally differentiated cells, immortalized cell lines, embryonic stem cells, and induced pluripotent stem cells generated through reprogramming of somatic cells. The latest step in this evolutionary continuum is the direct reprogramming of somatic cells into other somatic cell types from the same or different germ layers. These approaches have been applied to the study of pathophysiological mechanisms, diagnosis, and therapy. Although several clinical trials have been conducted based on prior preclinical research, only a limited number of applications (e.g., blood transfusion or bone marrow transplantation) have become established clinical practices in regenerative medicine. Several clinical trials based on iPSC technologies have been conducted. More recently, the first commercialized iPSC-based products for clinical use have been approved for cardiac and nervous tissue regeneration [65]. In addition, genome-editing tools (CRISPR, TALENs, ZFNs) represent a complementary technological approach for the replacement of damaged tissues resulting from inherited defects. Bottom, the potential of direct cellular reprogramming for disease modeling and clinical applications in regenerative medicine and oncology is currently limited by several challenges, including the use of viral vectors (integrative methods), the low efficiency of non-integrative approaches, incomplete specialization of converted cells, and concerns regarding their long-term phenotypic stability.

Although DCR avoids the formation of iPSC, thereby reducing tumorigenesis risks, several challenges remain (Figure 1), including the low efficiency of the process and concerns regarding cell specification, as reprogrammed somatic cells often do not exhibit identical transcriptional and proteomic profiles compared with the target naïve host cells [3]. These are not the only challenges. The following sections provide a more in-depth discussion of the current limitations and unresolved issues in DCR.

*Low reprogramming efficacy*: A general limitation of direct reprogramming approaches is their relatively low conversion efficiency in vitro, although efficiency often improves when reprogramming is performed directly in vivo [62,66,67]. Reprogramming strategies based on viral vectors remain the most effective. However, reported efficiencies are highly variable and typically low, generally ranging from 2% to 20% [68]. For instance, approximately 1.5% and 5% of cardiac fibroblasts were reprogrammed into cardiomyocytes at 1 and 4 weeks after in vivo delivery, respectively [69]. Notably, an in vitro study reported that lentiviral delivery can substantially increase conversion rates, with efficiencies exceeding 80% in fibroblast-to-neuron reprogramming under specific conditions [70]. Alternatively, in vivo approaches using retroviral delivery, which selectively infect dividing cells, can in some cases achieve high conversion efficiencies. For example, in vivo reprogramming of reactive glial cells into neurons has achieved conversion rates above 90% among infected astrocytes [66]. Lower efficiencies have been described for non-integrating viral systems such as Sendai vectors.

While in vitro systems provide controlled environments that enable systematic optimization of reprogramming cocktails and delivery methods, in vivo settings may favor partial reprogramming trajectories that are stabilized by niche-specific signals (metabolic, mechanical, electrophysiological) that are difficult to recapitulate in vitro. Mechanistically, in vivo reprogramming benefits from several factors. These include (i) the native extracellular matrix (ECM), whose composition and stiffness regulate mechanotransduction pathways such as YAP/TAZ signaling; (ii) paracrine signaling from neighboring cells, including immune and stromal populations, as well as dynamic inflammatory responses that may transiently increase cellular plasticity; and (iii) physiological oxygen gradients and metabolic conditions that influence epigenetic remodeling.

In relation to the latter, an often overlooked parameter is the discrepancy between physiological and in vitro oxygen levels, which may significantly impact both reprogramming efficiency and cellular behavior. For example, hematopoietic stem cells reside in relatively hypoxic niches in vivo, whereas standard in vitro culture is typically performed under atmospheric oxygen conditions. Exposure to ambient oxygen during cell isolation and processing can induce extra-physiological oxygen shock/stress, leading to increased mitochondrial ROS production and promoting differentiation at the expense of stemness [71]. In the context of cellular reprogramming, it has been shown that hypoxic conditions (~5% O_2_) enhance the efficiency of iPSC generation from human and mouse fibroblasts [72]. Similarly, reduced oxygen levels (~2% O_2_) have been reported to increase the efficiency of direct reprogramming of dermal fibroblasts into cardiomyocyte-like cells using Gata4, Mef2c, and Tbx5 [73]. These findings suggest that supraphysiological oxygen levels in vitro may limit reprogramming efficiency by promoting oxidative stress, reducing cellular plasticity, and impairing epigenetic remodeling. In line with this, modulation of redox balance, for example, through antioxidant treatments, has been shown to enhance the in vivo conversion of glial cells into neurons in a traumatic brain injury model [74], further underscoring the role of metabolic state as a determinant of reprogramming outcomes.

Another critical aspect that complicates direct comparisons is that in vitro and in vivo reprogramming efficiencies are often quantified using different metrics. In vitro studies typically report conversion efficiency relative to the total cell population exposed to reprogramming factors. In contrast, in vivo studies often report efficiencies relative to transduced cells. Importantly, these transduced populations frequently consist of reactive or proliferative cells that are already primed for plasticity, which may contribute to higher observed conversion rates, as exemplified by Guo et al. [66]. Therefore, rather than representing conflicting outcomes, in vitro and in vivo efficiencies reflect distinct but complementary dimensions of reprogramming performance. This distinction highlights the need for standardized metrics that integrate both conversion efficiency and target population coverage when evaluating reprogramming strategies.

*Determinants of reprogramming specificity and phenotypic stability*: Direct reprogramming frequently generates transcriptomic profiles that do not fully recapitulate those of their endogenous counterparts [62]. This incomplete fidelity is not only an intrinsic limitation of the reprogramming process but is also strongly influenced by the origin of the starting somatic cells, which determines both efficiency and outcome. For example, fibroblasts derived from different mouse strains give rise to induced neural stem cells (iNSCs) with variations in marker expression, transgene silencing, survival, and differentiation potential [75]. Similarly, fibroblasts derived from cardiac tissue are more amenable to cardiac reprogramming than those from the skin, supporting a role for epigenetic memory in shaping cell fate outcomes [76].

At a broader level, these sources of variability are further compounded by fundamental interspecies differences, as not all direct reprogramming techniques developed in mice are equally effective in human cells. For instance, the transcription factors Gata4, Mef2c, and Tbx5 (GMT) are sufficient to generate cardiomyocyte-like cells from mouse fibroblasts but fail to do so in human cells [77]. A well-known example illustrating these interspecies differences is that somatic cells from mice can typically be reprogrammed within one to two weeks, whereas human cells often require more time and generally exhibit lower reprogramming efficiency [78].

These interspecies discrepancies have been linked to differences in chromatin organization and transcription factor targeting. Comparative epigenomic analyses have shown that, although reprogramming factors recognize similar DNA motifs and target overlapping gene categories in mouse and human cells, their genome-wide binding landscapes diverge substantially. In particular, factors such as Oct4, Sox2, and Klf4 exhibit a stronger tendency to engage closed chromatin regions in human cells, whereas in mouse they preferentially target accessible enhancer elements [78]. In practical terms, this means that in human cells, reprogramming factors frequently encounter chromatin regions that are not readily accessible and must first induce local chromatin remodeling to enable gene activation. In contrast, in mouse cells, these factors more often act on pre-existing accessible regulatory regions, facilitating a more efficient and rapid initiation of the reprogramming process.

Together, these findings indicate that human cells impose a more restrictive epigenetic barrier to lineage conversion, requiring additional remodeling steps and contributing to the reduced efficiency, slower kinetics, and lower fidelity of reprogramming observed in human systems. Consequently, results obtained in rodent models may overestimate the robustness and translational potential of reprogramming strategies when applied to human cells. Addressing this gap would require reprogramming strategies specifically adapted to the more restrictive epigenetic landscape of human cells. Mechanistically, several studies have demonstrated that pioneer transcription factors such as NeuroD1 can directly engage repressed chromatin regions and actively induce local chromatin remodeling, including histone demethylation and acetylation, thereby enabling gene activation and cell fate conversion [79]. Ascl1 has also been identified as a pioneer transcription factor capable of accessing and remodeling closed chromatin at neuronal loci, thereby enabling gene activation. Another factor, the histone reader PHF7, in conjunction with the SWI/SNF complex, improved the reprogramming efficiency of fibroblasts into cardiac cells by enhancing chromatin accessibility and facilitating transcription factor binding at these sites [80]. In contrast, factors such as Brn2 and Myt1l act downstream, binding to these newly accessible regions to further stabilize and reinforce the neuronal transcriptional program [81]. In parallel, epigenetic modulators, including inhibitors of histone deacetylation or DNA methylation, can markedly enhance reprogramming efficiency [82]. Consistently, targeting specific chromatin-modifying enzymes can further influence lineage conversion outcomes: inhibition of the PRC2-associated histone methyltransferase EZH2 has been reported to improve cardiac reprogramming, whereas loss of the histone methyltransferase KMT2B severely impairs neuronal reprogramming [81].

Closely related to these issues of specificity is the question of phenotypic stability. Most studies have evaluated the stability of cellular reprogramming over short periods, typically for a few weeks. For example, the conversion of fibroblasts into functional neurons has been reported to remain stable for at least four weeks [83], while the reprogramming of pancreas-derived mature exocrine cells can yield stable β-cells that remain stable in vivo for at least two months [84]. However, the persistence and functional stability of reprogrammed cells beyond these time frames, and whether the induced cell fate is truly irreversible in the long term, remain largely unexplored. These outcomes will likely depend on the specific reprogramming strategy and the target cell type.

A critical factor linking specificity and stability is the extent of epigenetic remodeling achieved during reprogramming. Accumulating evidence indicates that incomplete erasure of donor cell epigenetic marks can influence transcriptional programs and compromise lineage stabilization. This phenomenon, well described in iPSC, is increasingly recognized in DCR, where residual DNA methylation patterns, histone modifications, and chromatin accessibility states may bias lineage outcomes or predispose cells to phenotypic instability. In other words, reprogrammed cells may retain epigenetic signatures reflective of their source tissue. A clear example of the functional consequences of epigenetic memory has been demonstrated in iPSC, where cells derived from different somatic sources exhibit distinct lineage biases linked to residual DNA methylation patterns. For example, fibroblast- or neural progenitor-derived iPSC retain hypermethylation at hematopoietic loci and show reduced blood differentiation potential, whereas blood-derived iPSC preserve epigenetic features (e.g., Wnt3 methylation) associated with enhanced hematopoietic output [85]. Similarly, early-passage iPSC retain a transient epigenetic and transcriptional memory of their cell of origin, which biases their differentiation propensity toward the donor lineage, an effect that is progressively erased upon extended passaging [86].

Consistent with this, single-cell RNA sequencing studies have revealed that reprogramming trajectories are often heterogeneous and can diverge toward alternative fates. For instance, during intermediate stages of fibroblast-to-neuron conversion, the neuronal differentiation trajectory can be disrupted by competing myogenic programs [87]. In this example, cells pass through a transient intermediate state marked by partial activation of neural and progenitor-associated genes (e.g., Sox9, Hes1), without fully committing to a canonical lineage. Accordingly, lineage fidelity remains a major challenge, as reprogrammed cells may fail to fully recapitulate the molecular and functional identity of their in vivo counterparts. Suboptimal factor combinations, stochastic gene expression, and incomplete chromatin remodeling can result in cells that fail to achieve full maturation [88]. In one study, direct reprogramming of fibroblasts into neurons using Ascl1, Brn2, and Myt1l induced rapid activation of neuronal gene expression (e.g., Tuj1, MAP2, synapsin), and converted cells were capable of firing action potentials and forming functional synapses. However, conversion remained incomplete, with efficiencies typically below 20%, indicating that only a subset of cells fully stabilizes the neuronal transcriptional program [59]. Notably, Ascl1 alone was sufficient to initiate partial neuronal features, but additional factors are required to suppress fibroblast identity and reinforce lineage fidelity. These observations suggest that lineage commitment during reprogramming depends on both activation of target programs and repression of the donor cell identity.

In vivo reprogramming may partially overcome these limitations by providing environmental cues that promote phenotypic stabilization. However, direct evidence demonstrating enhanced epigenetic consolidation compared to in vitro systems remains limited. Moreover, even in these settings, long-term lineage stability and the potential for phenotypic drift or reversion remain insufficiently characterized. Persistent expression of exogenous reprogramming factors or incomplete transgene silencing may further compromise lineage fidelity and functional maturation.

*Metabolic reprogramming as a determinant of cell fate conversion efficiency*: Direct lineage conversion requires not only the activation of new gene expression programs but also the establishment of a metabolic state compatible with the target cell identity. Importantly, these metabolic transitions are not merely downstream consequences of fate conversion but actively interact with the epigenetic landscape. Key metabolites, including acetyl-CoA, α-ketoglutarate, and S-adenosylmethionine, serve as cofactors for chromatin-modifying enzymes, thereby linking cellular metabolism to histone modifications and DNA methylation dynamics [89]. As a result, metabolic constraints can directly influence chromatin accessibility and transcriptional reconfiguration during reprogramming.

This functional role of metabolism is, for example, supported by comparative transcriptomic analyses showing that directly reprogrammed cardiomyocytes (iCMs) preferentially activate oxidative metabolic pathways, including fatty acid oxidation, whereas iPSC-derived cardiomyocytes rely predominantly on glycolysis, consistent with a less mature metabolic state [90]. These findings suggest that acquisition of an oxidative metabolic profile is associated with cardiomyocyte maturation during reprogramming, reinforcing the idea that metabolic state is a determinant of functional cell identity.

Recent work further refines this concept by showing that metabolic reprogramming during cardiomyocyte differentiation not only supports energy production but also promotes adaptive resistance to oxidative stress [91]. Specifically, the transition from glycolytic to oxidative metabolism is accompanied by increased mitochondrial activity and reactive oxygen species (ROS) production, yet differentiated cardiomyocytes exhibit enhanced antioxidant capacity and improved DNA damage repair, resulting in greater resilience to oxidative challenges. These findings indicate that successful lineage conversion requires not only metabolic rewiring but also the acquisition of protective mechanisms that enable survival and function in an oxidative tissue environment. A similar metabolic shift is observed in neuronal reprogramming, where increased mitochondrial mass and activity, driven by metabolic regulators such as PGC-1α and ERRγ, support the transition from aerobic glycolysis in neural progenitors to oxidative phosphorylation in mature neurons [92]. However, this transition also introduces vulnerabilities. Induced neurons derived from aged fibroblasts exhibit impaired mitochondrial function, including reduced oxidative phosphorylation (OXPHOS) gene expression, decreased mitochondrial membrane potential, and lower ATP production, revealing bioenergetic deficits that become evident upon the shift to oxidative metabolism [93]. More broadly, fibroblasts from aged individuals display mitochondrial dysfunction and elevated ROS levels that are retained during reprogramming and correlate with reduced efficiency and incomplete maturation [93]. Consistently, the shift toward oxidative metabolism during reprogramming is often accompanied by ROS accumulation and lipid peroxidation, which can compromise cell survival and reduce conversion efficiency. Modulating redox homeostasis can partially overcome these barriers. For example, co-expression of Bcl-2 or treatment with ferroptosis inhibitors such as liproxstatin-1 or vitamin E derivatives enhances neuronal reprogramming by reducing lipid peroxidation and activating antioxidative stress responses, thereby facilitating successful fate transition [74].

Collectively, these findings support a model in which metabolic state acts as a functional constraint during reprogramming. The acquisition of an oxidative, OXPHOS-dependent identity is necessary for lineage specification but simultaneously imposes bioenergetic and redox challenges that can limit efficiency, delay maturation, or reduce lineage fidelity. Therefore, integrating metabolic and redox modulation strategies with transcription factor delivery and epigenetic remodeling represents a key avenue to enhance the robustness and translational potential of direct reprogramming approaches.

*Functional integration of reprogrammed cells*: A further point of debate concerns the extent to which ex vivo-reprogrammed cells can effectively integrate into host tissues after transplantation, and how such integration might proceed when reprogramming is induced directly in vivo. In tissues such as the heart [47] or the pancreas [84], integration of induced cells involves comparatively lower architectural and functional complexity than in the brain. In contrast, functional integration in the central nervous system requires not only appropriate cell identity but also the establishment of synaptic connectivity, electrophysiological maturation, and incorporation into existing complex neural circuits.

Indeed, with the exception of species such as zebrafish, certain amphibians, and planarians, current evidence indicates that true brain regeneration does not occur in humans after injury. When partial recovery is observed, particularly following small lesions, it is generally attributed to the adaptive (and sometimes maladaptive) remodeling of perilesional regions [94]. Across human studies and most experimental stroke models, cortical reorganization is predominantly observed in motor representations adjacent to the lesion site [95,96].

Experimental approaches, including intracortical microstimulation in animal models and transcranial magnetic stimulation in humans, have provided evidence for this adaptive reorganization, reproducing patterns associated with spontaneous recovery or rehabilitation-driven improvements [95,97,98,99]. In rodent stroke models targeting the somatosensory forelimb area, functional changes emerge with a delay, as sensory-related activity gradually appears in peri-lesional motor regions weeks after injury. Notably, this temporal progression parallels the recovery of sensorimotor function. In line with this, we previously demonstrated in a mouse model of focal stroke affecting the forepaw somatosensory cortex that treatment with MSCs encapsulated in silk fibroin hydrogels led to the re-emergence of sensory function in adjacent motor areas 4–8 weeks after treatment, as assessed by somatosensory evoked potentials [100].

Therefore, a key uncertainty is whether reprogrammed cells, particularly those derived from glial scar conversion, can acquire the appropriate subtype identity and achieve functional integration at the circuit level. Functional integration of newly generated neurons has been supported by a growing body of preclinical studies combining structural, electrophysiological, and circuit-level analyses. For example, in a focal stroke mouse model, transplantation of human neural stem cells combined with pro-survival factors such as 3K3A-APC, promoted partial reconstruction of disrupted cortical circuits, including the formation of new projections from the motor cortex to injured somatosensory regions and the establishment of synaptic contacts between host neurons and graft-derived cells, as indicated by the co-localization of synaptophysin and PSD-95 [48]. These structural changes were accompanied by functional improvements at the network level, as demonstrated by in vivo voltage-sensitive dye imaging, which revealed enhanced sensory-evoked cortical responses with increased amplitude and faster kinetics. At the single-cell level, two-photon calcium imaging of grafted cells expressing GCaMP further showed that a subset of transplanted cells exhibited stimulus-locked activity patterns consistent with neuronal responsiveness. However, it is important to note that functional recovery remained partial, with evoked responses not fully reaching control levels, and that only a fraction of grafted cells displayed clear functional coupling to host circuits. Complementing these findings, more recent work using FOXG1-expressing forebrain progenitors derived from human iPSC has provided converging evidence of integration at multiple levels in stroke-injured adult rat cortex [101]. Ultrastructural analyses confirmed the formation of bidirectional synaptic contacts between graft-derived neurons and host cells, predominantly excitatory. Whole-cell recordings further demonstrated that a large fraction of transplanted neurons acquired mature electrophysiological properties, including the ability to fire action potentials. At the circuit level, trans-synaptic rabies virus tracing revealed that grafted neurons received afferent inputs from multiple brain regions, including the thalamus and cortex, indicating their incorporation into long-range networks. In parallel, PET imaging showed increased synaptic density in the grafted regions, suggesting broader remodeling of local circuitry. These effects were associated with improved sensorimotor coordination and a reduced incidence of post-stroke seizures. Together, these multi-technological approaches provide compelling evidence that transplanted cells can functionally integrate into host neural circuits. However, some limitations remain, as the injured adult cortex appears to restrict the differentiation of grafted cells into inhibitory neurons [101].

Some in vivo direct reprogramming studies have also provided evidence that induced neurons can acquire mature electrophysiological properties and integrate into host circuits. For example, whole-cell recordings of NG2 glia-derived neurons performed at 5–12 weeks post-reprogramming (using the pro-neural factors Ascl1, Ngn2, and NeuroD1), showed neuronal maturation signs, including increased membrane capacitance, decreased input resistance, and hyperpolarized resting membrane potentials, reaching values comparable to endogenous neurons at later time points [102]. As early as 5 weeks, reprogrammed neurons exhibited repetitive action potentials with properties resembling those of fast-spiking interneurons. Importantly, spontaneous excitatory and inhibitory postsynaptic currents were present, and pharmacologically blocked using CNQX and picrotoxin, respectively, demonstrating that these cells receive synaptic inputs from both glutamatergic and GABAergic host neurons. However, this study did not assess long-range projections, output synaptic connectivity, or contribution to circuit function, leaving the extent of functional integration at the network level unresolved. In another example, NeuroD1-mediated in vivo reprogramming of reactive glial cells generated neurons that, by ~4 weeks post-infection, exhibited large voltage-gated Na^+^ and K^+^ currents and fired repetitive action potentials, while cortical slice recordings revealed robust spontaneous synaptic events as well as evoked postsynaptic responses, indicating that the converted neurons formed functional synaptic connections with surrounding host neurons [66].

Whether similar neural integration can occur in humans remains uncertain. Several additional unresolved issues deserve attention. One concerns the number of reprogrammed cells required to achieve sufficient integration and functional restoration within the target tissue. The threshold for meaningful recovery remains largely unknown and likely varies across organs, injury types, and the extent of tissue damage.

In contrast, studies in the cardiac field have provided more direct evidence of functional integration following in vivo reprogramming. For instance, fibroblast-to-cardiomyocyte conversion using defined transcription factors (e.g., Gata4, Mef2c, Tbx5 and Hand2) has been shown to generate induced cardiomyocyte-like cells that form connexin-43-positive gap junctions with neighboring cardiomyocytes and exhibit synchronized calcium transients, consistent with electrical coupling to the host myocardium [62,67]. Importantly, these reprogrammed cells contributed to impulse propagation and improved cardiac function after injury, providing direct evidence of functional integration at the tissue level. Notably, the relative permissiveness of cardiac tissue to functional integration is further supported by its closer translation to clinical application. Engineered heart muscle grafts derived from human iPSC-cardiomyocytes have been successfully transplanted in non-human primates and advanced to early-phase clinical testing in patients with heart failure, where graft survival, vascularization, and signs of electromechanical coupling with host myocardium have been reported, highlighting the feasibility of achieving functional tissue-level integration in the heart [47]. Recently, iPSC-based therapies for cardiac regeneration have entered clinical commercialization in Japan [65].

**Figure 2:**
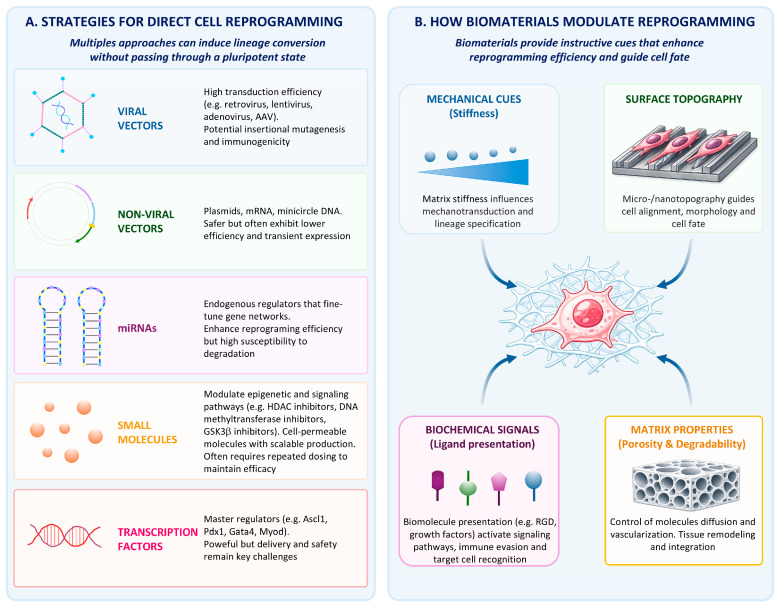
Biomaterial-based strategies for enhancing direct cell reprogramming. (**A**) Overview of the main approaches used for direct lineage conversion, including viral vectors, non-viral delivery systems, miRNAs, small molecules, and transcription factors. Each strategy presents distinct advantages and limitations regarding efficiency, safety, and cell fate stability. (**B**) Schematic representation of how biomaterials modulate cell reprogramming through multiple instructive cues. Mechanical properties (stiffness), surface topography, biochemical signaling, and matrix porosity/degradability influence mechanotransduction, cell morphology, molecular diffusion, vascularization, and tissue integration, thereby enhancing reprogramming efficiency and guiding cell fate specification.

Given the limitations outlined above, biomaterials can help enhance reprogramming strategies at both in vitro and in vivo scales and at various stages of the process. Engineered substrates that mimic features of the extracellular matrix, such as stiffness and topography, can improve reprogramming efficiency (Figure 2). Biomaterial-based systems can also facilitate the delivery of reprogramming agents, including viral and non-viral vectors, recombinant proteins, miRNAs, chemoattractants, or even angiogenic factors [103]. Precisely due to their ability to provide sustained, localized release of these factors over time, biomaterials may help stabilize induced cell fates, limit dispersion of reprogramming factors, reduce immune responses (particularly against viral vectors), and increase targeting specificity.

## 3. Biomaterial Applications: Classification and Relevant Properties

Over the past decades, major advances in biology and medicine, including deeper insights into stem cells and their niches, single-cell transcriptomics, and real-time in vivo imaging techniques such as multiphoton microscopy, have transformed our understanding of cellular behavior. In parallel, biomaterials have emerged as powerful tools for diagnosis and therapy, leveraging their ability to interface with cells and tissues. The convergence of biomedicine and materials science has been pivotal in developing applications ranging from surgical implants to drug delivery systems, impacting diverse fields such as vascular surgery and traumatology.

For both research and clinical applications, biomaterials can be sourced from natural components, including collagen, hyaluronic acid (HA), fibronectin, laminin, and silk fibroin [104], or from synthetic polymers such as polyethylene glycol (PEG), polylactic acid (PLA), titanium, and polyaryletherketones [105]. Natural biomaterials generally offer excellent biocompatibility, but this often comes with greater variability in production. In contrast, synthetic biomaterials provide more reproducible and tunable physicochemical properties. Natural and synthetic biomaterials have been employed in a wide range of biomedical applications, spanning from materials that provide structural functions and mechanical support to systems designed for drug delivery [106]. The scope of biomaterials is broad and versatile in biomedical research and clinical applications. Perhaps one of the most important developments has occurred in the area of cardiovascular surgery, aimed at preventing arterial restenosis after transluminal catheter angioplasty, through the use of classic metallic stents (stainless steel, cobalt-chromium, chromium-platinum) and drug-eluting stents with degradable polymers, an industrial field experiencing unstoppable growth [107]. Synthetic and biological heart valve prostheses are routinely used in clinical practice, both in conventional surgery and in transcatheter interventions [108,109]. The use of bioabsorbable patches to improve cardiac function after ischemia appears also promising. However, some injectable bioabsorbable scaffolds (IK-5001), based on alginate and calcium gluconate failed to prevent the adverse effects of ventricular remodeling in patients with myocardial infarction [110]. Another highly relevant field is the development of metallic and non-metallic prostheses, including titanium or polymer-based systems such as polyetheretherketone (PEEK), for the treatment of bone fractures and bone reconstruction in orthopedic, traumatological, and craniofacial surgery [111,112]. In the field of the nervous system, the role of biomaterials based on poly[bis(p-carboxyphenoxy)propane-co-sebacate] has also been demonstrated for the sustained release of the antitumor agent carmustine in the treatment of brain cancer [113]. Additional applications include nerve guidance conduits for peripheral nerve regeneration, shunts for the treatment of hydrocephalus, and implantable electrodes and neuroprosthetic devices.

These applications, among many others, rely on the current ability to design biomaterials with different geometries and formats, such as films, hydrogels, scaffolds, nanoparticles, fibers, and elastomers, for example [114,115,116]. Many engineered biocompatible materials can be produced in a wide range of formulations, including variations in polymer concentration, stiffness, crosslinking density, surface topography, geometry, secondary structure, and biodegradability. Although the modulation of these parameters provides flexibility for the development of diverse biomaterials, it also introduces significant challenges in achieving reproducible and predictable physiological responses. Given the importance of these parameters for the release of reprogramming molecules and the modulation of reprogramming efficiency, the following paragraphs provide an overview of these properties, their fabrication and control methods, and their impact on molecular release, cell viability, and differentiation.

### 3.1. Stiffness

**Figure 3:**
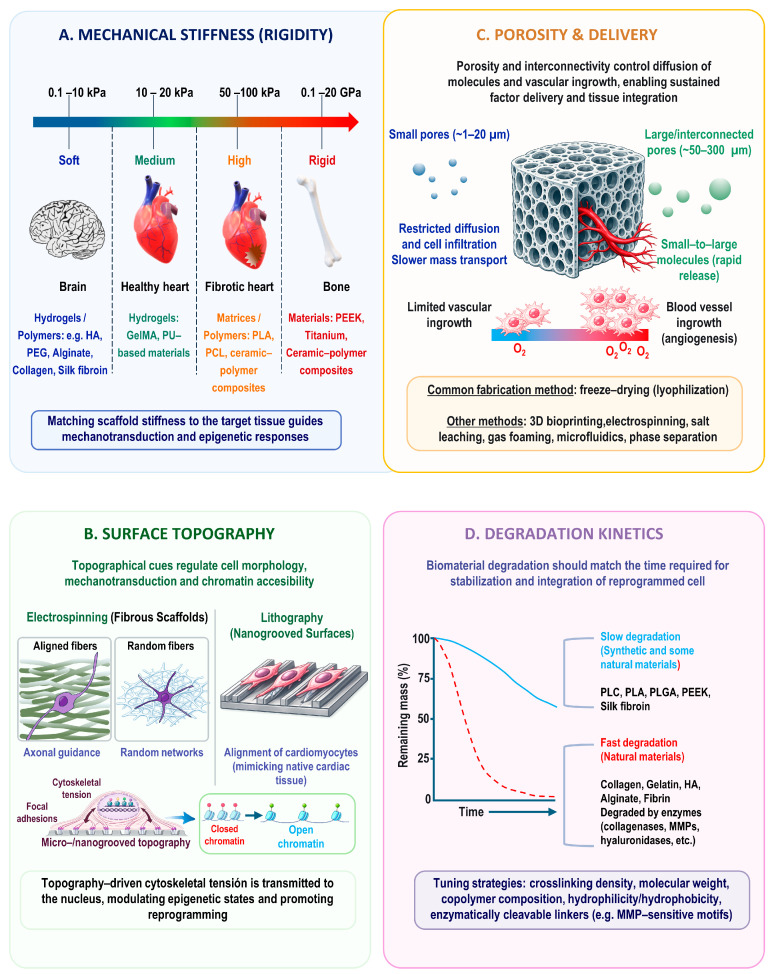
Key biomaterial design parameters influencing direct cell reprogramming. (**A**) Mechanical stiffness of biomaterials regulates mechanotransduction pathways, epigenetic responses, and lineage specification. Matching scaffold rigidity to the target tissue microenvironment can enhance reprogramming efficiency and functional maturation. (**B**) Surface topography, including aligned fibers and nanopatterned surfaces, modulates cell morphology, cytoskeletal organization, chromatin accessibility, and cellular alignment, thereby influencing lineage commitment and maturation. (**C**) Scaffold porosity and pore interconnectivity control molecular diffusion, nutrient transport, oxygen availability, cell infiltration, vascular ingrowth, and sustained delivery of reprogramming factors. Pore size also determines the balance between factor retention and release kinetics. (**D**) Biomaterial degradation kinetics must be synchronized with the timeline of reprogramming and tissue remodeling.

Beyond providing structural support, the physicochemical properties of biomaterials may influence cell reprogramming efficiency, maturation, and cell fate. As will be discussed later with specific examples, the elastic modulus of the target tissue is a key factor to consider in the design of any scaffold to support DCR. While the brain is one of the softest tissues in the body (~0.1–10 kPa) [117], other target tissues, such as the heart or bone, present significantly higher stiffness values (Figure 3).

These values can even be variable, as occurs in the myocardium under pathological conditions. Indeed, the adult heart has an elastic modulus of approximately 10–20 kPa, but this value can increase to 50–100 kPa or more in the fibrotic scar after a myocardial infarction [118]. In contrast, bone represents the upper limit of the biological mechanical spectrum. Bone reaches a Young’s modulus in the range of approximately 0.1–20 GPa, a value that varies across different studies and testing methods [119]. Overall, reprogramming efficiency improves when cells are cultured on substrates whose stiffness matches that of the target tissue. For example, soft hydrogels such as HA, alginate, PEG, collagen, or silk fibroin are ideal for brain applications, while stiffer hydrogels such as GelMA or polyurethane are better suited for the heart. The combination of synthetic polymers with robust mechanical properties, such as Polycaprolactone (PCL) or Polylactic Acid (PLA), together with natural or decellularized matrices, is ideal for both ex vivo and in situ reprogramming of the fibrotic heart. For bone regeneration, rigid materials such as metallic alloys, thermoplastics like PEEK, or ceramic-polymer composites are the most suitable.

The mechanical properties of a biomaterial can be modulated by changing different parameters, which reshape the scaffold architecture network. For example, changing the polymer concentration increases the mass density per unit volume and reduces the free volume between polymer chains. For most hydrogel systems, higher polymer concentrations usually yield stiffer networks. For instance, our group found that increasing the SF content from 2% to 6% dramatically enhanced the elastic modulus, regardless of the scaffold format (i.e., hydrogels or films) [39,120]. This rule applies to other biomaterials such as collagen or HA, commonly employed in applications with soft tissues. Even physiologically, an increase in collagen I concentration has been shown to increase ECM stiffness, enhancing stem cell multipotency [121].

Polymer concentration is not the only factor capable of tuning stiffness. Molecular weight (MW) and crosslinking density (related to the mesh size of the network) are also main determinants of stiffness, as both directly define the resulting network architecture. However, their influence on the mechanical properties of biomaterials is complex and often dependent on the scaffold system or platform. Higher molecular weight (MW) polymers, consisting of long polymer chains, typically exhibit increased stiffness due to enhanced chain entanglement, which acts as physical crosslinking and improves resistance to deformation.

The swelling capacity of a hydrogel is closely related to its network structure, particularly crosslinking density, which also influences stiffness. Higher water uptake is typically associated with a lower crosslinking density, allowing greater network expansion. In contrast, increased crosslinking restricts chain mobility and limits network swelling, resulting in reduced water uptake and higher stiffness. In some cases, it has been possible to dissect how these factors act independently and contribute differently to the overall mechanical properties through their effects on network architecture. For example, in non-crosslinked HA, swelling generally increases with MW, as longer polymer chains can retain more water. In contrast, in chemically modified or crosslinked hydrogels, the effect of molecular weight on swelling is system-dependent, as higher MW may either increase or decrease swelling depending on the balance between chain entanglement, intramolecular interactions, and effective network mesh size that restricts or promotes network expansion [122]. Thus, the effect of molecular weight on hydrogel stiffness is often non-linear. Increasing molecular weight may initially increase stiffness due to greater chain entanglement; however, at higher values, longer chains can reduce the effective crosslinking density, leading to increased swelling and decreased stiffness [123]. Although hydrogel swelling is generally inversely related to crosslinking density, mechanical properties can vary depending on molecular weight, polymer charge, intermolecular interactions, and the composition of the biomaterial. For example, collagen stiffness can be increased by adding HA, which helps reorganize collagen fibers and modify the matrix microstructure [124].

Since mechanical properties can be tuned through crosslinking, multiple strategies have been developed to modulate network density and reorganize the biomaterial microarchitecture. These strategies are based on chemical, physical, or hybrid strategies.

Chemical crosslinking is the most widely used approach, as it allows direct control over the number of covalent bonds within the polymer network. For example, methacrylated hydrogels such as GelMA or Polyethylene Glycol Diacrylate (PEGDA) can be crosslinked through photopolymerization, where the stiffness can be tuned by photoinitiator concentration or UV exposure time [125]. In another study, a biocompatible light-based azide-alkyne chemistry was used for spatiotemporal control of PEG hydrogels, tuning the stiffness and degradation of this biomaterial in contact with mesenchymal stem cells and 3T3 fibroblasts [126]. Chemical crosslinkers can also be used to modulate stiffness in natural biomaterials. For instance, genipin, glutaraldehyde, or carbodiimide (EDC/NHS) crosslinking have been widely used in collagen or gelatin scaffolds. Sundararaghavan et al. showed that genipin-crosslinked collagen hydrogels exhibited significantly higher mechanical stiffness [127]. Paradoxically, EDC/NHS crosslinking stabilizes collagen scaffolds through covalent bonding, but its effect on mechanical stiffness is highly dependent on fibrillar architecture and can range from no change to a reduction in high-strain modulus depending on crosslink localization [128,129].

In contrast, physical crosslinking relies on non-covalent molecular interactions such as ionic bonds, hydrogen bonding, and hydrophobic interactions. These systems often produce viscoelastic materials that more closely mimic the mechanical properties of native tissues. A well-known example is alginate, where divalent cations such as Ca^2+^ induce hydrogel formation through ionic crosslinking. The resulting stiffness can be tuned by controlling calcium concentration and gelation conditions [130,131]. More advanced strategies include interpenetrating polymer networks, in which two polymer networks are independently crosslinked, either simultaneously or sequentially, to achieve improved and more tunable mechanical properties. For example, Khetan et al. demonstrated that in covalently crosslinked methacrylated hyaluronic acid (MeHA) hydrogels, stem cell fate is regulated by cell-mediated matrix degradation and the resulting traction forces, rather than initial bulk stiffness [132]. In another study, EDC crosslinking was used to fabricate porous collagen-HA matrices, resulting in enhanced mechanical stability, improved resistance to collagenase-mediated degradation, and good compatibility with fibroblast cell lines [133].

Finally, enzymatically crosslinked hydrogels provide another powerful strategy to regulate stiffness under mild physiological conditions. For example, a phenol-functionalized gelatin was fabricated through carbodiimide-mediated conjugation of tyramine, enabling enzymatic gelation under physiological conditions via horseradish peroxidase (HRP) and H_2_O_2_. By adjusting the phenolic group content and reaction conditions (HRP and H_2_O_2_ concentrations), gelation time, mechanical properties, and degradability could be tuned [134].

### 3.2. Topography

Beyond mechanical stiffness, substrate topography is a key biophysical cue that regulates cell morphology, cytoskeletal organization, and ultimately DCR efficiency. Cells are highly sensitive to micro- and nanoscale surface features such as roughness, aligned grooves, micropillars, nanofibers, and hierarchical architectures. These topographical properties regulate adhesion, polarity, and cytoskeletal tension, thereby modulating mechanotransduction pathways including YAP/TAZ signaling, which has been linked to somatic cell reprogramming toward stem-like states. In fact, ECM remodeling has been proposed as a strategy to control YAP/TAZ activity, as this pathway integrates mechanical inputs such as matrix stiffness and regulates downstream transcriptional programs including TGF-β, BMP, and Wnt signaling [135]. As will be discussed in more detail later, biomaterial topography has been suggested to influence epigenetic regulation during cell reprogramming through mechanotransduction-mediated changes in nuclear organization and chromatin accessibility. Micro- and nanoscale surface features can modulate cytoskeletal tension and nuclear mechanics, which have been associated with changes in histone modification patterns. These epigenetic alterations are generally linked to a more permissive chromatin state that facilitates transcriptional activation and may contribute to enhanced reprogramming efficiency [68].

Among the most widely used approaches to modulate biomaterial surfaces are lithography-based patterning, electrospinning, 3D printing, soft lithography, and laser-based fabrication techniques.

Lithography-based patterning techniques, such as photolithography and electron-beam lithography, allow the fabrication of highly defined micro- and nanopatterns including grooves, ridges, pillars, and wells. In photolithography, a photosensitive polymer (photoresist) is spin-coated onto a substrate such as silicon or glass. Then, a photomask containing the desired pattern is aligned over the coated substrate and exposed to UV light. These patterns can then be transferred to biomaterials either directly or through secondary molding steps. Electron-beam lithography follows a similar principle but uses a focused electron beam instead of UV light. Complementarily, soft lithography is commonly performed by first fabricating a master mold using photolithography. A liquid elastomer, typically PDMS (polydimethylsiloxane), is then poured over the master mold and cured to form a flexible stamp. This stamp can be used to replicate micro- and nanotopographies onto hydrogels or polymeric materials by molding, microcontact printing, or replica molding. The process allows pattern transfer onto soft biomaterials such as collagen, gelatin, PEG, or HA hydrogels. Using these principles to produce nanopatterns in collagen it was possible to transdifferentiate human mesenchymal stem cells into neural-like cells [136]. In another example, PEG-based substrates with nanogroove features aligned neonatal rat ventricular myocytes in a manner resembling native cardiac tissue, leading to anisotropic organization and spontaneous contraction after two days in culture [137].

Electrospinning generates fibrous scaffolds by applying a high voltage between a polymer solution and a grounded collector. The electric field induces the formation of a charged polymer jet that stretches and solidifies into fibers as solvent evaporates. By adjusting parameters such as polymer concentration, solvent system, voltage, flow rate, and needle-to-collector distance, fiber diameter and surface roughness can be controlled. Additionally, collector design influences fiber organization: rotating drum collectors produce aligned fibers, while static collectors generate random networks. Electrospinning also allows control over nanoscale topography, which is particularly relevant for mechanotransduction. Advanced electrospinning setups, such as coaxial electrospinning, can also produce core-shell fibers with additional topographical complexity. One particularly relevant application of these principles is axonal guidance. For instance, aligned PCL nanofibers have been reported to promote neural differentiation of pluripotent embryonic stem cells while directing neurite extension [138]. Our group observed comparable outcomes using straining flow spinning (SFS), a technique inspired by the natural fiber formation processes of silkworms and spiders, which enabled axonal promotion and directional growth in primary cortical neurons [139].

3D printing and bioprinting enable topographical control through layer-by-layer deposition of biomaterials. Techniques such as extrusion-based printing deposit polymer filaments with defined spacing, creating surface features and controlled microarchitectures. Resolution depends on nozzle diameter, extrusion pressure, and printing speed. Other approaches, such as stereolithography (SLA) or digital light processing (DLP), use light-induced polymerization of photosensitive resins to generate complex microstructures with higher resolution. Several studies have shown that 3D-printed scaffolds with controlled microarchitecture and hierarchical porosity can modulate stem cell behavior, differentiation, and tissue formation by tuning both mechanical and structural cues [140,141,142].

Finally, Laser-based fabrication techniques include laser ablation and two-photon polymerization. Laser ablation uses focused laser pulses to selectively remove material from a surface, generating grooves, pits, or roughness patterns. Parameters such as laser power, pulse duration, and scanning speed determine feature size and morphology. An example of the utility of these technologies is the use of two-photon polymerization to fabricate biomimetic scaffolds with submicron resolution, enabling precise control over cell adhesion, alignment, and morphology through highly defined micro- and nanoscale topographies [143,144].

Each of these techniques presents distinct advantages and limitations in controlling biomaterial topography. Lithography-based methods offer extremely high resolution and reproducibility, enabling precise micro- and nanopatterning, but they are typically limited to planar substrates and require specialized facilities. Soft lithography provides a more versatile and cost-effective alternative, allowing pattern transfer onto soft biomaterials, although feature fidelity may decrease for very complex structures. Electrospinning enables the fabrication of ECM-like fibrous architectures with nanoscale control and high surface area, but pore size and pattern uniformity are more difficult to precisely regulate. In contrast, 3D printing allows spatial control of architecture across multiple length scales and fabrication of complex geometries, although its resolution is generally lower than lithographic techniques. Laser-based fabrication offers high precision and the ability to generate hierarchical and 3D microstructures, but it often involves slower processing times and higher equipment costs. Although these technologies enable precise control over biomaterial topography and introduce mechanobiological cues that regulate cell survival, adhesion, and differentiation, as well as modulate cellular signaling at proteomic and genomic levels, a full understanding of how substrate-mediated regulation enhances reprogramming efficiency and stabilizes cell fate in direct cellular reprogramming (DCR) remains limited.

### 3.3. Porosity

The porosity of a biomaterial and the connectivity of its internal architecture are critical aspects to consider when designing biomaterials as delivery systems for reprogramming factors and agents. Moreover, when biomaterials are intended to serve as carriers for reprogrammed cells that act as interfaces with the target tissue, the presence of pores and interconnected pathways becomes essential. These features enable the exchange of oxygen and carbon dioxide, nutrients, and metabolic waste between the interior and exterior of the material (Figure 3). Due to oxygen diffusion limitations, particularly in thick biomaterials, interconnectivity should be sufficiently extensive and wide to support vascular ingrowth and remodeling within the scaffold, as well as integration with the host tissue vasculature. An optimal balance between porosity, pore interconnectivity, and tortuosity is essential to regulate release kinetics and maintain the biological activity of reprogramming factors, at least during the time window required for cell conversion, which usually involves periods extending beyond several weeks [83,145]. Highly porous scaffolds typically allow faster diffusion and release of encapsulated molecules, whereas lower porosity or more tortuous pathways can slow diffusion and promote sustained release.

A wide set of fabrication strategies have been used to precisely control scaffold porosity. Electrospinning is one of the most widely used techniques to generate scaffolds with tunable pore size and architecture. Electrospun scaffolds exhibit high surface area and interconnected porous networks that can be optimized for controlled release applications. For example, a recent study developed electrospun nanofibrous scaffolds incorporating liposome-based gene delivery systems, demonstrating sustained BMP-2 gene release and enhanced osteogenic differentiation due to the controlled pore architecture and density of silk fibroin nanofibers [146]. In another study, electrospun scaffolds composed of PLA and polyvinyl alcohol (PVA) significantly increased pore size and overall porosity. This strategy improved wettability and reduced water absorption time, enhancing macrophage and keratinocyte infiltration, which may be relevant for skin tissue engineering applications [147].

Another widely used method to generate porous scaffolds is gas foaming, which produces highly porous structures through gas bubble formation within polymer matrices. Typically, biodegradable polymers such as poly(lactic-co-glycolic acid) (PLGA), PLA, or PCL can be exposed to high-pressure CO_2_, followed by rapid depressurization to nucleate gas bubbles and form porous structures. For example, in a pioneering study, highly porous PLGA scaffolds were fabricated using a combination of CO_2_ gas foaming and salt (NaCl) particulate leaching. In this case, pore size and scaffold architecture could be tuned by varying the salt-to-polymer ratio and the salt particle size [148]. Similarly, the combination of gas foaming and particulate (salt) leaching has been used to enhance pore interconnectivity and mechanical performance in poly(ε-caprolactone)/poly(ethylene oxide) (PCL/PEO) scaffolds. In this system, increasing the PEO content led to a decrease in pore size and an increase in pore density, while human endothelial cells tended to align along the direction of pore orientation [149].

Freeze-drying (lyophilization) is another widely used technique to generate porous hydrogels. During freezing, ice crystal formation determines pore size and distribution. Slower freezing rates produce larger pores, whereas rapid freezing leads to smaller pore structures. A mechanistic study has shed light on this process, which was investigated in a mixed pullulan-dextran hydrogel system [150]. After subjecting the hydrogel to a supercooling step, the initially formed ice grows into crystals that fracture within the polymer network, triggering secondary nucleation and resulting in a fine ice structure. As freezing proceeds, the polymer is expelled into the spaces between ice crystals, forming a continuous network upon thawing. The final pore size is determined by the ice crystal size, which decreases as the polymer network becomes more highly crosslinked.

Molecular weight, polymer concentration, and crosslinking density also influence scaffold porosity. Increasing crosslinking density typically reduces mesh size and pore dimensions, thereby limiting diffusion and slowing the release of encapsulated molecules. For example, PEG hydrogels with different crosslinking densities have shown significant differences in albumin diffusion and accumulation [151]. Our group recently showed that increasing silk fibroin concentration from 2% to 6% is sufficient to dramatically reduce the release of the chemokine SDF-1α (MW ~8.0 kDa). However, for small molecules such as the neurotransmitter acetylcholine (MW ~0.15 kDa), no significant changes in release rate were observed, suggesting that higher polymer content and crosslinking density can limit the diffusion of medium-sized biomolecules while having a negligible effect on very small molecules [39]. Similarly, molecular weight influences pore structure by modulating polymer chain length and entanglement. Higher molecular weight polymers generally form networks with larger mesh sizes prior to crosslinking, which can increase porosity and swelling capacity. However, as anticipated above, the situation becomes more complex when additional parameters are involved, as in crosslinked systems, where higher molecular weight can also increase chain entanglement and reduce effective pore size, depending on polymer concentration and the crosslinking mechanism.

### 3.4. Degradability

For a wide range of biomedical applications, controlling biomaterial degradation is recognized as a key parameter. In the context of DCR, this control is particularly important, as it influences the kinetics of reprogramming factor release, as well as the stability of the converted cells and their interaction with the host tissue. Biomaterial degradation can be driven by chemical and biological processes, leading to surface erosion, mass loss, changes in molecular weight, and alterations in mechanical and structural properties. Monitoring these processes is commonly performed in the field using a variety of physicochemical and mechanical characterization techniques [152].

In general, degradation of biomaterials might increase pore size and interconnectivity due to progressive polymer chain cleavage, allowing progressive release of encapsulated molecules. This may be particularly beneficial for DCR applications and sequential delivery of reprogramming factors. Ideally, biomaterials should subsequently degrade once cellular conversion and stabilization of the new phenotype have occurred. However, in some applications, long-term structural stability may instead be desirable, particularly in load-bearing tissues such as bone.

Natural biomaterials such as collagen, gelatin, fibrin, HA, and alginate typically undergo rapid degradation driven by enzymatic activity. For instance, collagen-based scaffolds are readily cleaved by collagenases, leading to progressive loss of stiffness and increased porosity over time, an effect that can be further amplified under inflammatory conditions. In contrast, silk fibroin is a natural biomaterial that surprisingly exhibits comparatively higher stability and resistance to degradation, likely due to its particular backbone structure and amino acid sequence, which lacks abundant recognition sites for common physiological proteases [153]. In contrast, synthetic polymers, including PCL, PLA, and PLGA, generally exhibit slower degradation rates, enabling prolonged structural support. Although polymers such as PCL can degrade through hydrolysis of ester linkages, their high crystallinity and hydrophobicity significantly limit water penetration into the bulk material, thereby slowing the overall degradation process [154]. For this reason, biomaterials can be designed to become more susceptible to chemical or biological breakdown, for example, through the incorporation of protease-sensitive motifs. Likewise, strategies that increase material hydrophilicity or reduce the degree of crosslinking, thereby promoting water uptake, are expected to lead to faster degradation rates. For example, electrospun PCL scaffolds can persist for months to years owing to their hydrophobicity and semicrystalline structure, whereas PLGA degrades at significantly faster rates that can be finely tuned by adjusting the lactic-to-glycolic acid ratio [154]. In a comparative study of electrospun PCL and PLGA scaffolds, PCL constructs produced after 90 min of electrospinning showed a slower degradation profile than PLGA scaffolds fabricated under the same conditions [155]. In another interesting study, two natural (collagen type II and chitosan) and two synthetic (PCL and PLGA) biomaterials were compared in their ability to support cartilage regeneration. It was observed that the natural scaffolds were almost completely degraded after 8 weeks post-implantation in the groin region, whereas degradation of PCL and PLGA remained incomplete [156].

Several strategies have been developed to precisely tune scaffold degradation: (i) One of the most widely used approaches is the modulation of crosslinking density. Increasing crosslinking density typically reduces water penetration and slows hydrolytic degradation. A recent study developed PEG-gelatin hybrid hydrogels with tunable crosslinking degrees between four-arm PEG-succinimidyl glutarate (4-arm PEG-SG) and gelatin. The resulting gelatin-PEG composite hydrogels demonstrated that higher crosslinking significantly slowed degradation and prolonged drug release [157]; (ii) Another approach involves modifying polymer molecular weight (MW), which affects degradation kinetics. Higher molecular weight polymers generally degrade more slowly due to reduced chain-end density and slower hydrolysis; (iii) Copolymerization and polymer blending are widely used strategies to modulate degradation behavior. For example, incorporating PEG functionalized with poly(L-lactide-co-ε-caprolactone) into PLLA matrices can enhance hydrophilicity, which facilitates water penetration and leads to a faster degradation rate. This accelerated degradation was accompanied by a progressive decline in both strength and stiffness [158]; (iv) It has also been demonstrated that post-functionalization strategies can be used to modify degradation rates. For example, a recent study modified PCL-based copolymers with N-acetylcysteine (NAC) via a thiol-ene reaction, which enhanced their degradation in the presence of the enzyme lipase. This effect was attributed to the increased hydrophilicity (as reflected by a decrease in water contact angle) following NAC functionalization, which facilitated water penetration into the polymer matrix [159]; (v) Another interesting strategy involves incorporating enzymatically cleavable linkers, for example, sequences sensitive to matrix metalloproteinases (MMPs). Several studies in the literature have demonstrated the potential of this approach, particularly in inflammatory environments, where there is typically an upregulation of MMP expression and secretion by inflammatory cells such as macrophages and neutrophils. A recent study designed a nanofiber hydrogel system in which the release of the angiogenic and neuroprotective peptide QK, an active fragment of VEGF that binds directly to VEGF receptors, was regulated through MMP-2-cleavable TIMP-derived sequences and amphiphilic molecules. These functional hydrogels enabled the on-demand release of QK in response to elevated MMP-2 levels in the injured brain tissue, promoting neuronal survival and improvement of motor function after brain injury [160]. Similar principles have been applied in the cardiovascular field. For instance, using Sulfo-SMCC chemistry, a myocardial infarction-responsive hydrogel based on glutathione (GSH)-modified collagen was developed to enable MMP-triggered release of basic fibroblast growth factor (bFGF). The system incorporated a recombinant GST-bFGF fusion protein linked through an MMP-2/9-cleavable peptide (PLGLAG). Following myocardial infarction in rats, the upregulation of MMP-2 and MMP-9 promoted hydrogel degradation and triggered on-demand bFGF delivery [161]. This strategy demonstrated improved cardiac function, enhanced vascularization, and attenuation of adverse myocardial remodeling. Although the effect of incorporating the MMP-responsive system on left ventricular ejection fraction was relatively moderate compared to non-responsive hydrogels, the impact on fibrosis was particularly significant, with approximately a 50% reduction in collagen deposition in the infarcted tissue. It would also be interesting to investigate the potential of these MMP-responsive constructs to promote regeneration in established lesions associated with persistent inflammatory components, where tissue repair may require the prolonged release of trophic factors.

Collectively, the controlled delivery of viral vectors and reprogramming factors likely represents an important application of biomaterial systems. Properties such as porosity, hydrophobicity, charge, the degree of crosslinking, the presence of tortuous escape pathways, and degradation kinetics must be carefully considered according to the desired spatiotemporal dynamics. For example, natural biomaterials that degrade rapidly in vivo, such as collagen, may restrict the release of reprogramming factors to just a few hours or days, compared to natural materials with high resistance to degradation, such as silk fibroin [153]. Scaffolds with high polymer concentration and crosslinking density, with pores smaller than 100 nm and tortuous release pathways, may hinder the release of viral factors, but not of recombinant proteins or microRNAs. Other parameters, such as charge and hydrophobicity, can also influence release behavior. However, the challenge lies in finding the perfect balance between reprogramming factor retention and release. For example, an excessively high net positive charge in the biomaterial can promote the retention of negatively charged microRNAs, whereas an excessively high negative charge can cause strong electrostatic repulsion and rapid release [162]. Perhaps the key lies in designing biomaterials with extreme properties that exert opposing effects; for example, in the case of microRNAs, using negatively charged biomaterials with tortuous escape pathways generated by a combination of polymer concentration and crosslinking density, or incorporating delayed degradability.

Most of these biomaterial properties might also influence another promising application of biomaterials: acting as an interface between reprogrammed cells and the target host tissue. Properties such as material stiffness, the degree of crosslinking, low-porosity scaffolds and tortuous escape pathways can, in turn, affect the integrity and functionality of these interfaces. Excessive mechanical compression and limited diffusion of oxygen and nutrients, especially in the inner regions of large scaffolds, particularly if they lack internal vasculature, might compromise the viability of reprogrammed cells. Restricted porosity can also limit cell-cell contact within the scaffold itself or between the scaffold and the host tissue. Pores exceeding 100 µm in diameter are typically conducive to vascular ingrowth [163]. In some applications, delayed degradation, which allows reprogrammed cells to establish initial contacts with host tissue, may promote subsequent electrical and metabolic integration with the host. Mechanical compatibility is also essential during the specific pathological conditions of the target tissue [164,165], not only for the cells encapsulated within the scaffold but also to avoid excessive compression of the host tissue, which could compromise subsequent phases of tissue remodeling.

In the following section, representative examples in the field of DCR are presented, highlighting the use of biomaterials, their fabrication methods, specific applications, and the biological responses observed.

## 4. Biomaterials for Direct Cell Reprogramming

### 4.1. Where Can Biomaterials Be Useful? What Challenges Can They Address in DCR?

Despite the great advances that cellular reprogramming has achieved in recent years, this technology still faces significant challenges for clinical translation. One of the most significant barriers is the need to efficiently develop in vivo reprogramming protocols capable of generating the desired somatic cell type without diverting differentiation toward unintended lineages. In this context, the sustained and controlled delivery of reprogramming factors, with precise regulation of their combination and concentration, may be essential to ensure efficient conversion and long-term stabilization of cell fate.

Clinical safety is another critical concern. As discussed above, most direct reprogramming approaches rely on the reintroduction and overexpression of transcription factors, encoding transgenes, typically delivered via viral vectors, most notable lentiviruses and retroviruses, which pose risks such as insertional mutagenesis and increased tumorigenicity, and complicate the production of fully differentiated, transgene-free cells. Non-integrating viral systems, such as adeno-associated viruses (AAV), adenovirus, or Sendai virus, offer safer alternatives, with AAV vectors being considered the gold standard and the preferred carrier for the delivery of reprogramming factors for clinical use, mainly because the transgene remains episomal [166]. However, important limitations remain, including restricted packaging capacity and the induction of both innate and adaptive immune responses [167,168].

At early stages, innate immune responses triggered by viral capsids or nucleic acids can reduce transduction efficiency and impair the initial activation of reprogramming programs. For example, Toll-like receptor 9 (TLR9) has been implicated in the recognition of vector-derived DNA, leading to the activation of NF-κB signaling and the production of pro-inflammatory cytokines, chemokines, and type I interferons, which can interfere with transduction and promote the development of adaptive immune responses [169]. However, these responses are highly vector-dependent and are generally more transient and less pronounced in AAV-based systems compared to other viral platforms. Interestingly, transient inflammatory signaling has, in some contexts, been associated with enhanced cellular plasticity, although excessive or sustained inflammation is generally detrimental. Notably, injury-induced reactive glial cells exhibit enhanced susceptibility to reprogramming in vivo [66], consistent with evidence that innate immune signaling promotes epigenetic remodeling and a more permissive chromatin state, thereby facilitating nuclear reprogramming [170].

At later stages, the emergence of antigen-specific immune responses leads to the presentation of capsid-derived peptides via MHC molecules, enabling CD8^+^ and CD4^+^ T cell recognition. Notably, this process does not depend on vector integration, as antigen presentation occurs even with non-integrating systems such as AAV. Consequently, cytotoxic T cell-mediated clearance of transduced cells, together with pre-existing neutralizing antibodies, can limit the efficacy of reprogramming [167,171]. These challenges define a clear opportunity for biomaterial-based approaches. By enabling localized and sustained delivery of viral vectors, biomaterials can reduce systemic exposure and thereby limit immune activation. For instance, hydrogel-based systems can spatially confine vectors within the target tissue, improving transduction efficiency while minimizing off-target distribution. In addition, biomaterials may provide partial physical shielding of viral particles, protecting them from neutralizing antibodies and proteolytic degradation, while also modulating release kinetics.

Biomaterials may also actively modulate the local immune microenvironment. For example, controlling the spatiotemporal presentation of vectors and bioactive molecules can reduce excessive inflammatory signaling and limit macrophage activation, thereby creating a more permissive niche for cell fate conversion and stabilization. In this context, chemically modified alginate microcapsules have been shown to attenuate the foreign body response by reducing the recruitment of macrophages, neutrophils, B cells, and CD8^+^ T cells, as well as limiting fibrotic overgrowth and collagen deposition at the implant site. This immunomodulatory effect preserves nutrient exchange and supports long-term cell viability, ultimately enabling sustained functional outcomes in immunocompetent models without the need for systemic immunosuppression [172]. In another example, PEG microgels decorated with factors targeting the programmed cell death-1 (PD-1) pathway have been used to control immunological rejection of transplanted pancreatic islets [173]. By immobilizing and prolonging the local availability of PD-L1, these biomaterials promote the expansion of FoxP3^+^ regulatory T cells, induce T cell anergy, and suppress effector immune responses. This localized, material-driven immune regulation significantly improves long-term graft survival (>100 days in ~60% of recipients) without the need for systemic immunosuppression. Although the immunomodulatory roles of biomaterials remain relatively underexplored in DCR, they represent a promising avenue to enhance both the efficiency and long-term stability of in vivo cell fate conversion.

Biomaterials can also act as carriers for the sustained release of non-viral reprogramming inducers over extended periods [174]. For example, without the introduction of ectopic transgenes, it has been possible to induce the in vitro conversion of human fibroblasts to functional neurons using a cocktail of 7 small chemical compounds, including valproic acid (VPA), forskolin, CHIR99021 (a GSK3 inhibitor) or Repsox (a selective inhibitor of the TGF-β type 1 receptor) among others [83]. These neurons were maintained and analyzed for up to 4 weeks, requiring the repeated administration of these molecules during the first two weeks for induction and further maturation. In another study, the same four factors (VPA, Repsox, forskolin, CHIR99021), together with additional ones, also contributed to the in vitro transformation of fibroblasts into functional cardiomyocytes capable of triggering action potentials [145]. In this case, at least three weeks of induction were required, with a constant supply of these factors every four days. These examples illustrate that safer, non-integrative approaches to DCR (e.g., plasmids, microRNAs, recombinant proteins, or small molecules) [175] require repeated administration of the delivered factors at effective concentrations over days to weeks to ensure efficient cell conversion.

In addition, many reprogramming factors have poor stability in both in vitro and in vivo settings. Most recombinant proteins exhibit limited stability in systemic circulation due to rapid proteolytic degradation and clearance, resulting in short half-lives that often range from minutes to hours unless chemically modified or otherwise protected. Moreover, denaturation of exogenously administered proteins can expose non-native conformations that are recognized as foreign by the immune system, thereby accelerating their clearance and degradation [176]. Similarly, microRNAs in their free form are highly vulnerable to degradation in vitro and, even more so, in vivo [177]. Thus, at first glance, the use of biomaterials could be highly relevant for preserving the biological activity of these intrinsically unstable reprogramming factors and for enabling better control over their delivery.

As an alternative to in situ DCR, which aims to inhibit reparative remodeling or convert fibrotic tissue into functional tissue, several promising DCR applications are based on the ex vivo generation of tissues and organs through the combination of reprogrammed cells with biomaterials such as films, hydrogels, or scaffolds. In this context, biomaterials play a crucial role by acting as structural and functional bridges between donor and host tissues.

### 4.2. Biomaterials: Current Approaches in the Field of DCR

Despite their promising potential, the use of biomaterials in cell reprogramming strategies remains largely unexplored. According to current literature, their application has mostly focused on a few key aspects.

**Figure 4:**
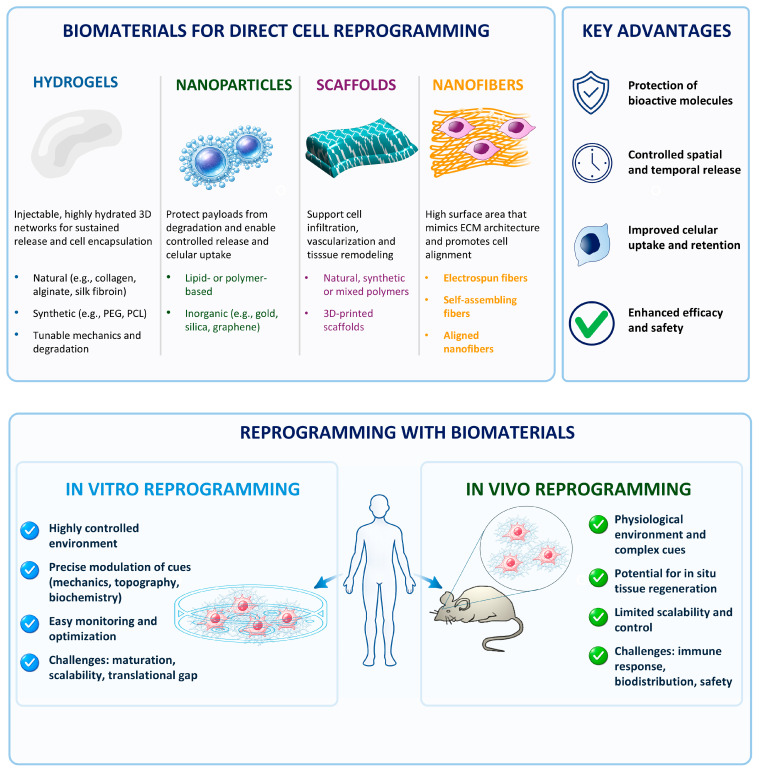
Current applications of biomaterials in direct cellular reprogramming. Different biomaterial systems, including hydrogels, nanoparticles, scaffolds, and nanofibers, have been developed to enhance direct cellular reprogramming by providing structural support, controlled delivery of reprogramming factors, and instructive biochemical and mechanical cues. Reprogramming approaches can be performed both in vitro, where environmental parameters are highly controllable, and in vivo, where physiological microenvironmental cues may promote tissue regeneration but also introduce additional translational and safety challenges. In vitro applications additionally include the generation of organoids for disease modeling and treatment validation, as well as the development of bioengineered tissue interfaces and constructs designed to enhance the structural and functional integration of reprogrammed cells following implantation in vivo.

First, in vitro, biomaterials can improve reprogramming protocols by modifying the substrate’s surface properties and topography. In vivo, biomaterials can restrict the release of reprogramming agents to the target area, thereby reducing the risk of systemic immune reactions. They can also function as protective carriers for reprogrammed cells and as supportive interfaces that promote integration with the host tissue (Figure 4). Moreover, biomaterials can be loaded with chemotactic factors to enhance cell retention and prevent graft dispersion. For instance, our group recently developed silk fibroin scaffolds for the controlled release of the chemotactic factor SDF-1α, which enhanced the retention of various stem cell types in nervous tissue [39].

To illustrate how these concepts have been applied, several representative studies in the field are discussed below (Table 1). The fabrication strategies, physicochemical properties, and functional roles in DCR are summarized in Table 2.

#### 4.2.1. Enhancing Reprogramming Efficiency Through Biomaterial-Driven Adhesion and Gene Delivery

As mentioned above, some studies have reported the use of biomaterials to enhance the efficiency of reprogramming protocols. For example, PEG has been shown to provide precise control over the adhesion of proteins and adhesion peptides to fibroblasts, leading to more efficient conversion into cardiomyocyte-like cells [178]. In this study, PEG-based hydrogels were fabricated via Michael-type addition reactions between multi-arm PEG derivatives (PEG8-VS and PEG8-Am) under mildly basic conditions, followed by surface functionalization with extracellular matrix proteins (e.g., Matrigel or laminin) or adhesion peptides such as RGD to modulate cell-material interactions.

It is well known that introducing genes into poorly adherent cells, such as hematopoietic cells, is challenging. However, in some cases, seeding hematopoietic cells onto culture plates coated with fibronectin or peptides derived from this extracellular matrix protein has been shown to considerably increase viral transduction efficiency [196]. In an interesting study, graphene oxide (GO)-Fe_3_O_4_-PEI complexes were used for non-viral (episomal) delivery of reprogramming factors into blood cells [190]. These nanocomposites were synthesized by first generating graphene oxide sheets via a modified Hummers’ method, followed by in situ deposition of Fe_3_O_4_ nanoparticles through chemical co-precipitation of iron salts under alkaline conditions. The resulting GO-Fe_3_O_4_ hybrids were subsequently functionalized with polyethyleneimine (PEI) to obtain a positively charged surface, enabling efficient binding and delivery of plasmid DNA. In combination with magnetic stirring and near-infrared (NIR) photothermal stimulation, the GO-Fe_3_O_4_-PEI complexes were able to reprogram these cells toward pluripotent stem cells with partial commitment to smooth muscle cells, cardiomyocytes, and endothelial cells [190]. Whether this strategy could be applied in vivo remains to be determined, given its potential for direct reprogramming of floating blood cells. Other non-viral gene carriers have also been employed; for example, cationic nanoparticles based on Porphyra yezoensis polysaccharide (PYP) were used for intracellular delivery of reprogramming factors, catalyzing the conversion of 3T6 fibroblast cell lines into neural cells within two weeks after transfection [191]. In this case, the polysaccharide was first chemically modified via periodate oxidation followed by ethylenediamine (Ed) conjugation to introduce cationic groups, yielding Ed-PYP. The resulting polymer was then assembled with plasmid DNA encoding reprogramming factors through electrostatic coacervation, forming nanoparticles capable of efficient gene delivery.

#### 4.2.2. Engineering Substrate Topography and Stiffness to Enhance Direct Cellular Reprogramming

**Figure 5:**
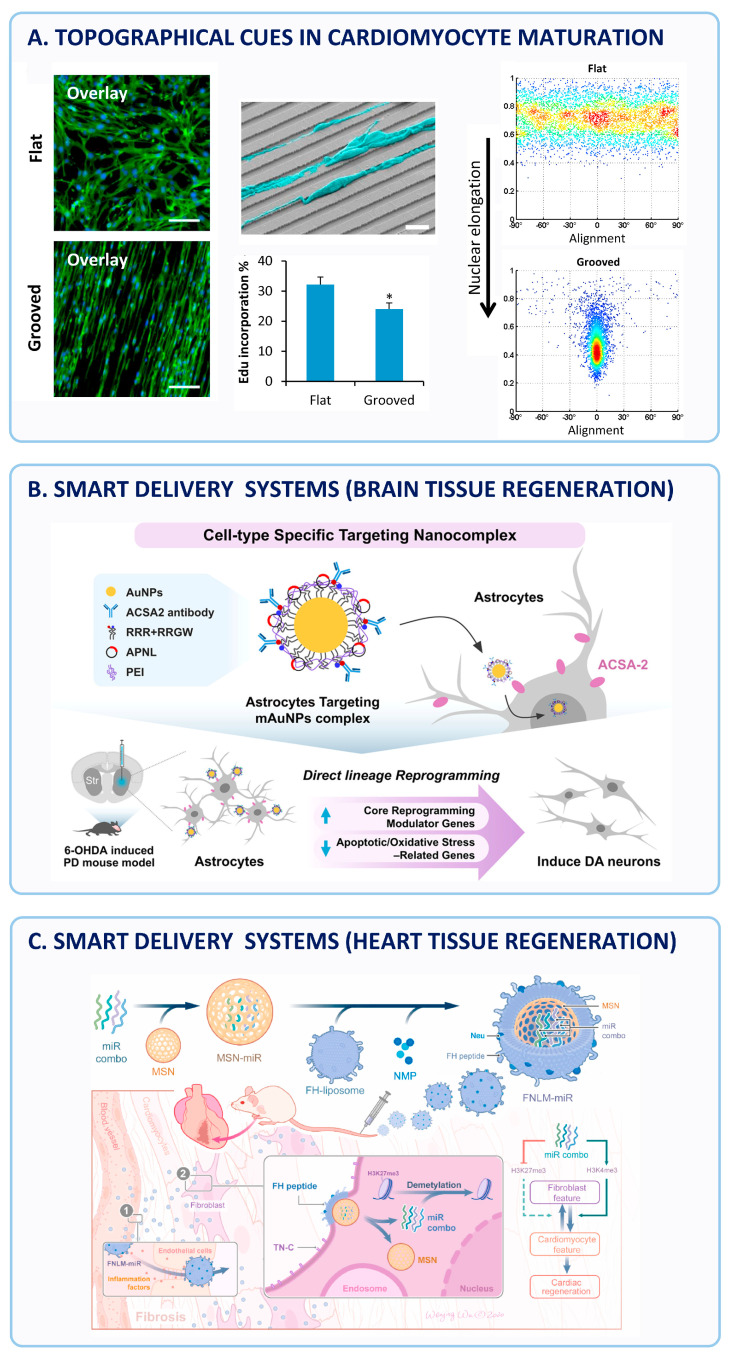
Representative examples of biomaterial-based strategies for direct cell reprogramming and tissue regeneration. (**A**) One of the key findings reported by Morez et al., illustrating how microgrooved topographical substrates enhance cardiomyocyte reprogramming and maturation by promoting cell alignment, nuclear elongation, histone H3 acetylation, and sarcomere organization. These biophysical cues mimic aspects of the native cardiac microenvironment and improve the efficiency of transcription factor-mediated cardiac lineage conversion (adapted from Morez et al., Biomaterials, 2015) [181]. (**B**) Cell-targeted gold nanoparticle nanocomplexes engineered for the selective in vivo reprogramming of astrocytes into dopaminergic neurons in a Parkinson’s disease model. Functionalized AuNP-based delivery systems enabled astrocyte-specific delivery of reprogramming factors (Ascl1, Pitx3, Nurr1, and Lmx1a), promoting efficient neuronal conversion, activation of pro-reprogramming epigenetic pathways, restoration of dopamine levels, and significant recovery of motor function (reproduced from Hwang et al., Acta Biomaterialia, 2026) [195]. (**C**) Biomimetic sequential-targeting nanoparticles engineered for the selective in vivo reprogramming of cardiac fibroblasts into induced cardiomyocyte-like cells after myocardial injury. Neutrophil membrane-coated mesoporous silica nanoparticles functionalized with FH peptide enabled sequential homing to inflamed myocardium and specific targeting of fibroblasts through tenascin-C recognition, allowing efficient intracellular delivery of cardiac reprogramming microRNAs (miR-1, miR-133a, miR-208, and miR-499). This strategy promoted direct cardiac reprogramming, reduced fibrosis, improved cardiac function, and demonstrated the therapeutic potential of non-viral precision nanomedicine for cardiac regeneration (reproduced from Wang et al., Biomaterials, 2021) [193].

Biomaterials offer vast opportunities for mechanical signaling, beyond merely delivering reprogramming factors, to optimize direct cellular reprogramming protocols. Changes in substrate topography have been shown to exert a strong influence on reprogramming efficiency [179,180]. For example, nanogrooved Polyurethane acrylate-based substrates induced epigenetic memory through histone modifications, which contributed to more efficient reprogramming of fibroblasts into neurons [180]. These substrates were fabricated using UV-assisted capillary force lithography, generating micro- and nanoscale groove patterns by varying the groove width (1.2 μm and 400 nm, respectively) while maintaining a constant ridge size. Scanning electron microscopy confirmed high-fidelity replication of the designed topographies. Following fabrication, the patterned surfaces were coated with gelatin to promote cell adhesion, enabling precise control over cell-substrate interactions through defined topographical cues. In another study, nanoscale surface topographies were combined with non-viral gene delivery systems to enhance fibroblast reprogramming efficiency into functional neurons capable of eliciting action potentials [68]. The polymeric carrier was based on poly(CBA-ABOL), synthesized via Michael polyaddition between N,N′-cystaminebisacrylamide (CBA) and 4-amino-1-butanol (ABOL), followed by purification through dialysis and structural confirmation by NMR spectroscopy. DNA nanocomplexes (polyplexes) were then formed by electrostatic self-assembly between the cationic polymer and plasmid DNA in HEPES-buffered conditions at optimized polymer-to-DNA ratios. In parallel, the cellular microenvironment was engineered using polydimethylsiloxane (PDMS) substrates fabricated via soft lithography from silanized master molds, enabling the generation of defined topographical patterns. The cured PDMS surfaces were subsequently plasma-treated, sterilized, and coated with fibronectin to enhance cell adhesion prior to seeding and transfection. Histone modifications, such as increased acetylation, and higher reprogramming efficiency of cardiac progenitors into cardiomyocyte-like cells have also been observed on microgrooved substrates fabricated on silicon wafers and replicated using polydimethylsiloxane (PDMS) [181] (Figure 5), consistent with improved fibroblast-to-cardiomyocyte reprogramming on the same microgrooved surfaces [182].

These microgrooved patterns (10 μm width and 3 μm depth) were generated using standard photolithography with SU-8 photoresist, followed by PDMS casting, curing, and peeling from the silicon master mold. The resulting substrates were plasma-treated and coated with extracellular matrix proteins (e.g., collagen I) to support cell adhesion prior to culture.

Although these examples can be useful for inducing direct in vitro reprogramming with the goal of transplanting converted cells or developing organ-on-chip systems for studying pathophysiological mechanisms, diagnostics, and drug testing, among other applications, it would be interesting to exploit the possible influence of surface topography on in situ reprogramming.

Not only substrate topography, but also substrate stiffness appears to contribute to reprogramming efficiency. It appears that reprogramming is more efficient when performed on substrates whose stiffness closely matches that of the target tissue. For example, higher efficiency in reprogramming murine fibroblasts into osteoblasts using Runx2/Dlx5 factors has been demonstrated on rigid (approximately 40 kPa) collagen-coated polyacrylamide hydrogels [183]. In this system, stiffness was tuned by adjusting the hydrogel composition, while collagen functionalization was used to promote cell adhesion and ensure effective mechanotransduction. In another example, chemical reprogramming of fibroblasts into functional neurons using non-integrative methods was much more effective on soft, collagen I-based substrates with stiffness close to that of brain tissue (~450 to 850 Pa) [184]. In this study, collagen type I hydrogels were prepared by neutralizing acidic collagen solutions under physiological conditions, followed by thermal gelation at 37 °C to form soft, three-dimensional substrates. The mechanical properties of the hydrogels were subsequently characterized by nanoindentation using a Hertzian contact model to determine the effective Young’s modulus. These soft, biomimetic substrates, further coated with gelatin to promote cell adhesion, provided a microenvironment favorable to neuronal conversion.

#### 4.2.3. Biomaterial-Based Microenvironments for Improving Stability and Function of Reprogrammed Cells

Other strategies have explored the use of biomaterials to create microenvironments that improve the viability and stability of reprogrammed cells. For example, HA/gelatin-based hydrogels have been used to immobilize spheroids composed of mature hepatocyte-like cells derived from direct reprogramming of fibroblasts, enabling the evaluation of drug toxicity and metabolic function [185]. In this study, these ECM-mimicking hydrogels were formulated by combining thiolated HA and gelatin with a polyethylene glycol diacrylate (PEGDA) crosslinker in the presence of a photoinitiator, followed by rapid photocrosslinking to encapsulate cell spheroids. These constructs were integrated into microfluidic platforms, providing a controlled 3D environment that supported tissue functionality and long-term cell viability. In another example, chitosan-g-oligo(L,L-lactide) (CLC) hydrogels were used to stimulate neural differentiation of progenitor neurons derived from human bone marrow-derived mononuclear cells [186]. The CLC copolymer was synthesized via mechanochemical grafting of chitosan with oligo(L,L-lactide), followed by the preparation of a photosensitive formulation containing polyethylene glycol diacrylate (PEGDA) and a photoinitiator. Hydrogels were then obtained through UV-induced photocrosslinking, and, in some cases, further structured into 3D scaffolds using two-photon stereolithography. These hydrogels could have applications in reconstructing nerve bundles, for instance, following spinal cord injury. In another example, biosheets of pullulan nanogels have also been used in combination with directly converted myoblasts to treat gastroschisis [187]. These nanogels were fabricated from cholesterol-bearing pullulan derivatives modified with acryloyl groups, which were subsequently crosslinked with thiolated PEG via chemical gelation. The resulting hydrogels were processed through freeze-thaw cycles and lyophilization to generate a porous structure, followed by collagen coating to enhance cell adhesion. In vivo, these implants resulted in improved differentiation, with the presence of multinucleated muscle-like cells and engraftment of skeletal muscle in a mouse model of gastroschisis, compared to the limited efficacy of Matrigel implants with converted myoblasts [187]. These same nanogels have been employed as supportive scaffolds for grafting directly converted osteoblasts derived from human fibroblasts [188]. In this case, NanoCliP-FD hydrogels were further functionalized with cell-adhesive molecules such as RGDC peptides or fibronectin. This biomaterial allowed efficient osteoblast adhesion, with the ability to produce calcified bone matrix and promote bone regeneration in a mouse model of bone defect. In another study, nanothin and nanoporous PLGA membranes were developed to facilitate contact between reprogrammed human fibroblasts and murine cardiomyocytes, creating a biomimetic cardiac microenvironment [189]. These membranes were fabricated by spin-coating PLGA solutions onto silicon substrates under controlled humidity conditions using a vapor-induced phase separation (VIPS) process, which enabled the formation of well-defined nanoscale porosity and ultrathin structures. The resulting membranes supported cell-cell interactions and paracrine signaling while maintaining physical separation, and their architecture remained stable during long-term culture. In fact, after four weeks, this co-culture combined with electrical stimulation substantially increased the efficiency of fibroblast-to-cardiomyocyte-like cell reprogramming, with cells expressing lineage-specific markers, although no contractile function was observed.

#### 4.2.4. From Passive Scaffolds to Smart Delivery Systems

Far fewer studies have employed materials as controlled-release systems for DCR. In an interesting study, polyethyleneimine (PEI)-based polyplexes have been employed to deliver microRNAs (miR-1 and miR-133a), significantly improving fibroblast-to-cardiomyocyte reprogramming efficiency when combined with fibronectin-functionalized poly-L-lactic acid (PLLA) electrospun scaffolds [192]. In this system, PLLA nanofibrous matrices were fabricated via electrospinning and subsequently functionalized with fibronectin. The PEI-miRNA complexes were immobilized onto the scaffold surface through physical adsorption, enabling localized and sustained release of microRNAs. Physicochemical characterization confirmed uniform fiber morphology, successful protein functionalization, and high miRNA loading efficiency, while release studies demonstrated controlled miRNA delivery over time. In a seminal study (Figure 5), Wang et al. [193] developed a sophisticated biomimetic nanoplatform for the in vivo delivery of microRNAs (miR-1, miR-133a, miR-208, miR-499) for the reprogramming of fibroblasts into functional myocardial tissue following cardiac injury. Mesoporous silicon nanoparticles (MSNs) were first engineered to efficiently encapsulate microRNAs using calcium silicate-based loading strategies, enabling high cargo capacity and sustained release. These cores were subsequently coated with a lipid bilayer via extrusion, forming a hybrid nanostructure, and further functionalized with a Tenascin-C-targeting FH peptide to promote selective binding to inflamed myocardial tissue. To evade detection by the circulatory system, these nanoparticles were additionally decorated with neutrophil-derived membrane proteins. Physicochemical characterization confirmed successful assembly, with stable nanoparticles (~170 nm) displaying positive surface zeta potential values (~10–15). Intravenous administration of these biomimetic nanoparticles led to improved cardiac function and attenuation of fibrosis in an ischemia-reperfusion mouse model. Importantly, this biomimetic coating reduced macrophage uptake and protected miRNAs from enzymatic degradation. This study represents one of the few examples illustrating the capacity of biomaterial-based systems for the delivery of reprogramming factors in vivo, using non-viral and non-integrative methods, in this case microRNAs, for better control of release and targeted local delivery of reprogramming factors to cardiac fibroblasts [193]. In another inspiring study, self-assembling peptide hydrogels, based on the IKVAV epitope present in laminin, have also been used to control the release of AAV-based viral vectors for delivery of the NeuroD1 transgene in brain tissue [194]. The peptide system (Fmoc-DDIKVAV) was engineered to undergo pH-triggered self-assembly into a nanofibrous hydrogel network, with tunable mechanical properties, mesh size, and high water content, all of which are critical parameters governing vector diffusion and release kinetics. Structural and rheological analyses confirmed the formation of a viscoelastic, shear-thinning matrix suitable for minimally invasive delivery. This strategy enabled controlled release of the viral vector in subcortical regions and the conversion of reactive astrocytes into neurons, reducing glial scarring in a mouse model of traumatic brain injury. This study highlights the potential of bioinstructive hydrogels not only as passive carriers but as active regulators of both spatial and temporal delivery of genetic reprogramming factors, enabling localized and sustained in vivo cell fate conversion within the central nervous system.

In a recent study (Figure 5), a lentiviral strategy based on functionalized gold nanoparticles was developed to achieve efficient in vitro and in vivo conversion of astrocytes into dopaminergic neurons [195]. The system allowed the delivery of reprogramming factors (Ascl1, Pitx3, Nurr1, and Lmx1a), specifically to astrocytes through antibody-mediated targeting based on the astrocytic marker ACSA2. The AuNP nanocomplexes were engineered via thiol-gold chemistry and PEGylation to avoid AuNP aggregation. The nanoparticles were also functionalized with a cell-penetrating peptide (RRR-PEG-SH), which enhances interaction with the cellular membrane and cellular uptake, and with an Fc-binding peptide (RRGW-PEG-SH) to ensure controlled antibody orientation and proper recognition of the ACSA2 marker. PEI was also incorporated to condense plasmid DNA and enhance intracellular delivery, promoting endosomal escape and improving transfection efficiency. In a Parkinson’s disease mouse model, striatal injection of these AuNPs led to increased generation of dopaminergic neurons, restoration of striatal dopamine levels, and significant improvements in motor function.

In summary, biomaterials offer diverse opportunities to enhance direct cellular reprogramming by modulating key microenvironmental cues, including substrate composition, stiffness, and surface topography. These approaches can improve reprogramming outcomes in both highly adherent and low-adherence cells, including challenging populations such as blood-derived cells. Despite these advances, most studies remain confined to in vitro settings, and substantial work is still required to optimize biomaterial-based platforms and translate them into clinically relevant applications. Thus, the in vivo landscape remains comparatively underexplored, likely due to the complexity of integrating reprogramming strategies with synthetic and natural biomaterials and of exploring the biological effects in a whole organism.

Although several studies have reported improved reprogramming outcomes using biomaterial-based systems, direct quantitative comparisons across studies remain challenging. Variability in donor cell type, target cell identity, reprogramming strategies, biomaterial composition and format, and efficiency metrics, ranging from marker-based estimates of conversion efficiency to functional maturation, limit cross-study comparability. In many cases, biomaterials modulate specific aspects of the reprogramming process, such as cell adhesion, cytoskeletal organization, mechanotransduction, gene delivery efficiency, or epigenetic remodeling. Where quantitative data are available, improvements are often modest but reproducible, typically on the order of ~2–5-fold increases in conversion yield for certain platforms, such as ECM-functionalized hydrogels or nanotopographical substrates. In contrast, other systems primarily enhance functional maturation, gene expression stability, or in vivo integration rather than absolute efficiency. Together, given the current state of the field, biomaterials should be viewed as context-dependent modulators of distinct stages of the reprogramming process rather than universal enhancers of conversion efficiency. In the relatively limited number of in vivo applications reported to date, their primary contribution often lies in protecting reprogramming factors or vectors from premature degradation, enabling localized and sustained delivery within the target tissue, and limiting off-target effects and inflammatory responses. Future studies incorporating standardized metrics and side-by-side comparisons will be essential to accurately quantify their impact and guide the rational design of biomaterial-assisted reprogramming strategies.

## 5. Outlooks

Where might cellular reprogramming lead us? To what extent are converted cells truly equivalent to their target counterparts, and can they integrate functionally within both simple and complex tissues? Will biomaterials become essential components for enhancing reprogramming efficiency and supporting the functional stability and long-term fate of reprogrammed cells? These questions lie at the frontier of our understanding of rapidly expanding and continuously evolving reprogramming technologies.

Over the past three decades, preclinical research has driven the development and characterization of a broad spectrum of biomaterials for diverse applications, including drug delivery, cell engraftment, and structural scaffolding in fields such as traumatology, neurology, and cardiovascular medicine. However, although biomaterials have been implemented in clinically relevant contexts, for example, in cardiovascular disease through drug-eluting stents, their use as delivery platforms for molecular factors, stem cells, or engineered cellular grafts has only partially entered the field of clinical regenerative medicine. A similar scenario exists in the field of cellular reprogramming. Despite more than two decades of intensive research, cellular reprogramming technologies remain largely confined to the preclinical stage. A notable exception is the recent approval and commercialization in Japan of allogeneic iPSC-derived therapies, including cardiomyocyte patches for the treatment of severe heart failure and dopaminergic neural progenitor cells for Parkinson’s disease. Nevertheless, these approvals have generated some skepticism within the scientific community due to the limited clinical trial data currently available [65]. Perhaps, given the still scarce clinical application of cellular reprogramming technologies, the translation of more complex strategies combining reprogramming and biomaterials will likely require even longer to reach clinical practice, mainly for two major reasons.

The first problem lies in the inherent limitations of cellular reprogramming technologies previously discussed. Among these, it is important to highlight once again the low efficiency of cellular conversion and the limited ability to generate cells with functional properties fully equivalent to those of native cells. In addition, concerns remain regarding genetic and epigenetic stability, as well as the substantial heterogeneity among the cellular populations generated during the reprogramming process. In contrast to iPSC-based reprogramming, in which both cellular identity and age-associated DNA methylation signatures are largely reset during the acquisition of pluripotency, direct reprogramming does not fully erase these features. This limitation may represent a disadvantage for regenerative applications aimed at rejuvenating aged or dysfunctional tissues, as directly reprogrammed cells can retain age-related epigenetic and functional alterations from the donor cells. However, the preservation of age-associated signatures may also be advantageous for disease modeling. Another limitation is that the optimal stoichiometry of the factors included in reprogramming cocktails remains poorly defined and often differs considerably among research groups. Consequently, it has become common for the same target cell type to be generated using multiple protocols and distinct combinations of reprogramming factors. For example, cardiomyocytes have been successfully generated from fibroblasts both in vitro and in vivo, either by overexpressing the transcription factors Gata4, Hand2, Mef2c, and Tbx5 [67], or by using microRNAs such as miR-1, miR-133, miR-208, and miR-499 [61]. In another example, ASCL1 alone was able to convert fibroblasts into functional neurons [197], and a similar outcome was achieved by inducing repression of the polypyrimidine-tract-binding (PTB) protein [198]. Another aspect discussed throughout this review concerns the greater refractoriness or resistance of human cells to reprogramming compared with somatic cells from other mammalian species, particularly mice. For example, the transcription factors cocktail GMT, commonly used to efficiently reprogram mouse fibroblasts into induced cardiomyocytes, shows substantially lower reprogramming efficiency in human cells [199]. This further emphasizes the need to adapt reprogramming protocols to clinically relevant human settings, including the development of biomaterial platforms capable of more faithfully recapitulating the human tissue microenvironment, for instance by providing human-like biomechanical and biochemical cues. Important safety concerns are also associated with the use of viral vectors. In general, the greater robustness and sustained transgene expression provided by retroviral and lentiviral vectors, which typically translate into higher reprogramming efficiencies, are counterbalanced by major safety issues, including insertional mutagenesis and tumorigenesis. The use of adenoviral or Sendai viral vectors, which are considered safer, is nevertheless limited by immunological rejection. Moreover, their transient transgene expression generally results in lower and less sustained reprogramming efficiencies compared with retroviral or lentiviral systems. The administration of immunosuppressive agents may help, but also raises important safety concerns, including an increased susceptibility to infections.

Secondly, the variability and heterogeneity inherent to direct cellular reprogramming also extend to the field of biomaterials. As discussed throughout this review, only a limited number of studies have preclinically demonstrated the ability of biomaterials to support or enhance in vivo reprogramming processes [188,193,194,195]. However, these strategies are typically complex and involve multistep fabrication procedures in which the biomaterial carrier is not only loaded with reprogramming factors, but is also engineered to selectively recognize, target, and release these factors within specific cell populations. Consequently, major concerns arise regarding how such sophisticated systems could be standardized and manufactured at scale for clinical applications. Supporting this notion, most biomaterials currently used in clinical practice (e.g., titanium implants, vascular stents) are based on relatively simple materials and manufacturing processes. The incorporation of bioactive molecules into these systems, such as antibodies or reprogramming-factor payloads, undoubtedly increases the complexity of production and complicates the level of standardization required for clinical translation and commercial approval. This challenge becomes even more significant when biomaterials are integrated with reprogramming factors or viral/non-viral gene delivery systems, since these combined platforms may fall within the category of ATMPs, which are subject to highly demanding regulatory, manufacturing, and safety requirements prior to clinical approval. Therefore, advancing this field will require greater standardization of both biomaterial fabrication and cellular reprogramming protocols, which would improve reproducibility across studies and facilitate direct comparisons between research groups to identify the most efficient and clinically translatable combinations of reprogramming strategies, biomaterial carriers, and manufacturing approaches for each pathological setting.

Probably due to its comparatively lower complexity, together with the more extensive understanding of cardiac pathophysiology, an increase in the clinical application of cellular reprogramming strategies in cardiac regenerative medicine is anticipated in the coming years, particularly through the use of iPSC-derived cardiomyocytes. This perspective is supported by the emergence of early clinical studies and the recent commercialization of iPSC-based cardiac therapies [47,65].

There is little doubt that regenerative medicine can greatly benefit from the enormous potential offered by biomaterials for both in vitro and in vivo direct cellular reprogramming. Just as biomaterials have already been extensively employed to support the delivery and engraftment of stem cells and differentiated cells across a wide range of pathologies, particularly in preclinical settings, they may also provide powerful platforms to enhance the efficiency, specificity, and safety of cellular reprogramming strategies (Table 3). Furthermore, recent advances in fourth-generation biomaterials have led to systems capable of sensing and responding to microenvironmental cues. For instance, photosensitive hydrogels functionalized with groups capable of absorbing photons can modulate the physicochemical properties of the material [200]. In another example, dynamic biomaterials responsive to pH variations have been developed for wound repair in type I diabetes, undergoing conformational changes (gel/sol states) that adapt dynamically to both pH and glucose levels [201]. Such 4D strategies, along with other emerging approaches, could confer unprecedented dynamism to the field of in vivo direct cellular reprogramming, enabling real-time adjustments of the reprogramming extent based on functional feedback from the target tissue.

Direct cellular reprogramming represents a fundamentally new approach to tissue regeneration and may, in turn, drive biomaterials science into previously unexplored territory. Biomaterials are expected to play a central role in cellular reprogramming by enabling immune modulation, controlled delivery of reprogramming factors and bioactive molecules, and the development of scaffolds capable of supporting the growth, maturation, and long-term stability of reprogrammed cells, either within damaged tissues or as part of engineered tissues and organs for transplantation.

## Figures and Tables

**Table 1:** Relevant studies utilizing biomaterials for direct cell reprogramming.

Biomaterial	Starting Cell Type/Target Cell Type/Reprogramming Factors	Engineering/Delivery Strategy	Main Outcomes	Ref.
PEG hydrogels	Mouse fibroblasts (adult/embryonic) >> cardiomyocyte-like cells Reprogramming: Fibroblasts obtained from mice carrying a doxycycline-inducible polycistronic OSKM cassette	PEG hydrogels covalently functionalized with Matrigel, laminin or RGD (variable concentrations)	In vitro: High-laminin/RGD PEG hydrogels doubled the yield of cardiomyocyte-like cells relative to Matrigel-coated TCP	[178]
PDMS	Mouse fibroblasts (embryonic) >> induced neurons Reprogramming: Lentiviral Vectors (factors: Ascl1, Brn2, Myt1L, M2rttA)	Micrograting topography (UV-photolithography)	In vitro: Higher number of neurons (Tuj+) and neurite outgrowth respect to smooth surfaces. Reprogramming efficiency ascribed to non-muscle myosin II (mechanical cue)	[179]
PUA	Mouse fibroblasts (embryonic) >> induced dopaminergic neurons Reprogramming: Lentiviral Vectors (factors: Ascl1, Pitx3, Nurr1, Lmx1a)	Microscale and nanoscale grooved patterns (UV-assisted capillary force lithography)	In vitro: Nanogrooved substrates doubled reprogramming efficiency compared with flat or microgrooved surfaces: dopaminergic markers functional maturation, and neuronal alignment/elongation. Enhanced efficiency linked to accelerated MET, cytoskeletal reorganization, and histone modifications	[180]
PDMS (topography changes)/CBA/ABOL (delivery of reprogramming factors)	Mouse fibroblasts (embryonic) >> induced neurons Reprogramming: Non-viral, DNA nanocomplexes (factors: Brn2, Ascl1, Myt1l)	Nanoscale patterns (UV-soft-lithography)	In vitro: Transfection efficiency remained unchanged across patterns. Hierarchical patterns increased reprogramming. Efficiency of neuronal conversion (higher number of neurons Tuj+/MAP2+) and significant neurite outgrowth	[68]
PDMS	Mouse cardiac progenitors >> cardiomyocyte-like cells Reprogramming: Lentiviral Vectors (factors: Myocardin, Tbx5, and Mef2c)	Micropatterned PDMS membranes (UV-soft lithography)	In vitro: Parallel microgrooves increased the number of functional cardiomyocyte-like cells, promoted cellular alignment, and induced a tenfold increase in sarcomere organization, compared with to flat substrates. Effects linked to increased histone H3 acetylation	[181]
PDMS	Mouse fibroblasts (neonatal) >> cardiomyocyte-like cells Reprogramming: Retroviral Vectors (factors: Gata4, Mef2c, Tbx5 and Mkl1)	Micropatterned PDMS membranes (UV-soft-lithography)	In vitro: Microgrooved substrates enhanced fibroblast-to-cardiomyocyte reprogramming, promoting sarcomere organization and spontaneous contractility. Microgrooves induced Mkl1 upregulation, which increased conversion efficiency	[182]
Collagen-coated polyacrylamide (PAM) hydrogels	Mouse fibroblasts (adult) >> osteoblastic-like cells Reprogramming: Non-viral vectors, plasmid transfection (Runx2, Dlx5)	Reprogramming on PAM hydrogels with stiffness matching collagenous bone substrate (~40 kPa) In vivo implantation of stiffness-primed reprogrammed cells encapsulated in collagen hydrogels in a mouse femur defect model.	In vitro: Efficient in vitro reprogramming on stiff, bone-like substrates; ROCK-related pathway activation drives fibroblasts toward osteoblast-like cells with high alkaline phosphatase, osteocalcin, Ca^2+^, and matrix mineralization In vivo: Four weeks after transplantation, X-ray imaging showed increased high-density mineralized bone. Marked improvement in defect repair	[183]
Col I hydrogels	Human skin fibroblasts (adult) >> induced neurons Reprogramming: Non-viral, small molecules cocktail (VPA, CHIR99021, Repsox, Forskolin, SP600625, GO6983, Y-27632, Dorsomorphin)	Hydrogels formed at 37 °C (>30 min) with stiffness matching brain tissue (450–850 Pa)	In vitro: Improved reprogramming versus TCP. Induced neuronal morphogenesis and markers and functional glutamatergic neurons. Col I hydrogels promoted MET via miR-615-3p-mediated ITGB4 regulation	[184]
HA/gelatin-based hydrogel and PEGDA	Human fibroblasts (embryonic/adult) >> hepatocyte-like cells Reprogramming: Lentiviral vectors (factors: hnf4a, foxa2, foxa3, atf5, prox1, and hnf1)	Hydrogel-based immobilization of 3D liver spheroids (hepatocytes/hepatocyte-like cells, Kupffer, stellate, and endothelial cells) integrated into a two-organ microfluidic system	In vitro: Validation of 3D liver spheroid viability and functionality, demonstrating proof of concept for liver cancer drug screening	[185]
Chitosan-g-oligo (L,L-lactide) copolymer hydrogel	Human bone marrow-derived mononuclear cells >> human neural progenitor cells Reprogramming: Non-viral vectors, plasmid transfection (factors: Msi1, Ngn2, and MBD2)	3D scaffolds produced by two-photon stereolithography	In vitro: Enhanced neuronal differentiation of neural progenitor cells seeded on the 3D scaffolds.	[186]
NanoCliP-FD nanogel (CHP-OA + PEGSH)	Human fibroblasts >> myoblasts Reprogramming: Retroviral vectors (factors: Myod1 and Mycl)	NanoCliP-FD nanogel: chemically crosslinked, freeze-thawed & freeze-dried, interconnected porous gel combined with converted myoblasts, placed on biosheet (autologous connective tissue), implanted in mouse gastroschisis model	In vivo: Enhanced differentiation of myoblasts into desmin- and myogenin-expressing muscle-like cells	[187]
NanoCliP-FD nanogel (CHP-OA + PEGSH)	Human fibroblasts >> osteoblast-like cells Reprogramming: Retroviral vectors (factors: Osterix, Oct3/4 and L-myc)	Nanogel coated with fibronectin to permit adhesion of osteoblast-like cells. Implantation in a mouse model of bone defect in femur	In vitro: Efficient adhesion and proliferation of osteoblast-like cells and production of calcified bone matrix on fibronectin-coated NanoCliP-FD gel. In vivo: Enhanced bone regeneration with formation of a large callus leaving small defect lesions	[188]
Nanothin and nanoporous PLGA membranes	Human fibroblasts (neonatal) >> cardiomyocyte-like cells Reprogramming: Non-viral vectors, plasmid transfection (factors: Gata4, Mef2c, Tbx5, Hand2, and Nkx2.5)	Cardiac-mimetic culture system: coculture of transfected fibroblasts and cardiomyocytes separated by a PLGA membrane, with application of external electrical stimulation	In vitro: Enhanced direct cardiac reprogramming (increasing levels of cardiac troponin T and other cardiac markers, but not contractile function)	[189]
Graphene oxide (GO)-Fe_3_O_4_-PEI complexes	Human PBMC (adult) >> iPSC committed to mesodermal lineages Reprogramming: Non-viral vectors, plasmid transfection (factors: Sox2, Klf4, L-Myc, Lin28, Oct3/4, and shRNA against p53)	Combined magnetic stirring and near-infrared laser irradiation to enhance transfection of suspension blood cells by increasing complex-cell contact and transiently improving membrane permeability	In vitro: Enables rapid PBMC reprogramming into partial iPSC (Nanog+/Oct4+/Sox2+). These cells efficiently transdifferentiate into mesodermal lineages without reaching full pluripotency (future application to generate muscle cells, cardiomyocytes, and vascular endothelial cells)	[190]
Cationic nanoparticles based on Ed-PYP	Mouse fibroblasts (embryonic; 3T6 cells) >> induced neural cells Reprogramming: Non-viral vectors, plasmid transfection (factors: Ascl1, Brn2 and FoxA1)	Cationized Porphyra yezoensis polysaccharide (Ed-PYP) used as a carrier to form nanoparticles with plasmids for gene delivery	In vitro: Lower cytotoxicity than Lipofectamine 2000 or polyethylenimin, with efficient gene delivery and conversion of fibroblasts into neural cells	[191]
Smooth and porous PLLA scaffolds	Human cardiac fibroblasts (adult) >> cardiomyocyte-like cells Reprogramming: Non-viral Vectors, direct transfection of miR1 and miR133a	Fibronectin-coated PLLA scaffolds loaded with PEI-miRNA polyplexes, with human fibroblasts seeded onto the scaffolds	In vitro: Enhanced reprogramming efficiency of PEI-miRNA polyplexes immobilized on fibronectin-coated PLLA scaffolds, enabling sustained miRNA delivery for ≥2 weeks. Porous scaffolds favored higher expression of cardiac markers (troponin T, GATA-4) in cardiomyocyte-like cells	[192]
Mesoporous silicon nanoparticles	Mouse cardiac fibroblasts (embryonic/adult) >> cardiomyocyte-like cells Reprogramming: Non-viral vectors, direct transfection of miR1, miR133, miR208, and miR499	Nanoparticles embedded in liposomes decorated with neutrophil membrane proteins and coated with FH peptide targeting Tenascin-C in inflammatory myocardium Minimally invasive intravenous injection of biomimetic nanoparticles, surface-decorated to target cardiac fibroblasts, delivering miRNAs	In vitro: 24 h treatment reprogrammed cardiac fibroblasts into functional cardiomyocyte-like cells, with increased cardiac gene expression, ion channels, sarcomeric markers, and beating activity; associated with histone H3 methylation/demethylation remodeling In vivo: Efficient targeting and reprogramming of cardiac fibroblasts in a mouse ischemia/reperfusion model, reduced fibrosis and improved cardiac function	[193]
Self-assembling peptide (SAP) hydrogel containing laminin epitope (IKVAV)	Mouse (postnatal) or human (embryonic) astrocytes >> induced neurons Reprogramming: Adeno-Associated Viral (AAV)Vectors (factor: NeuroD1)	SAP hydrogels for controlled AAV-NeuroD1 release. Intracerebral injection of SAP hydrogel into the glial scar 10 days after needle-stick injury.	In vitro: Efficient reprogramming, induced neurons express specific neural markers and are functional (action potentials) In vivo: Reprogramming of endogenous reactive astrocytes with hydrogel confined to the injection site, leading to reduced glial scar, and attenuation of astrogliosis and microglial activation	[194]
AuNP nanocomplexes	Mouse (primary astrocytes, in vitro; adult astrocytes, in vivo) >> induced dopaminergic neurons (iDA) Reprogramming: Lentiviral vectors (factors: Ascl1, Pitx3, Nurr1, and Lmx1a)	AuNP nanocomplexes engineered via thiol-gold chemistry and PEGylation. Functionalized with a cell-penetrating (RRR-PEG-SH) and Fc-binding (RRGW-PEG-SH) peptides to ensure antibody orientation and recognition of ACSA2 marker and cellular uptake.	In vitro: Efficient reprogramming of astrocytes into dopaminergic neurons, with expression of neuronal and dopaminergic markers (Tuj1, TH, DAT, VMAT2) and functional dopamine production (functional maturation) In vivo: Increasing dopaminergic neuron numbers, restoration of dopamine levels, and significant improvement in motor function in a Parkinson’s disease mouse model	[195]

Table abbreviations: PEG: Poly(ethylene glycol); OSKM: Oct4, Sox2, Klf4, and c-Myc; TCP: tissue culture polystyrene; PDMS: Sylgard 184 poly(dimethylsiloxane); PUA: Polyurethane acrylate; MET: mesenchymal-to-epithelial transition; CBA: N,N-cystaminebisacrylamide; ABOL: 4-amino-1-butanol; Col I: type I collagen; HA: hyaluronic acid; PEGDA: polyethylene glycol diacrylate; CHP-OA: acryloyl group-modified cholesterol-bearing pullulan; PEGSH: pentaerythritol tetra(mercaptoethyl) polyoxyethylene; PLGA: poly(lactic-co-glycolic acid); PEI: polyethylenimine; PBMC: peripheral blood mononuclear cells; iPSC: induced pluripotent stem cells; Ed-PYP: ethylenediamine-modified Porphyra yezoensis polysaccharide; PLLA: poly-L-lactic acid; AAV: adeno-associated virus.

**Table 2:** Examples of main biomaterial platforms used in DCR, their fabrication strategies, physicochemical properties, and functional roles.

Functional Category	Biomaterial Systems (Examples)	Fabrication/Design Strategy	Key Properties	Material-Driven Advantages	Limitations	Ref.
Surface-engineered substrates (adhesion control)	PEG hydrogels, fibronectin-coated substrates	Michael-type addition (PEG8-VS/PEG8-Am), ECM/peptide functionalization (RGD, laminin)	Tunable surface chemistry, controlled ligand density, low protein adsorption (PEG)	Precise control over cell adhesion via ligand presentation; antifouling background reduces nonspecific interactions; reproducible chemistry	Limited intrinsic bioactivity (requires functionalization); bulk PEG lacks degradability unless modified	[178,196]
Non-viral gene delivery nanocarriers	GO-Fe_3_O_4_-PEI, Ed-PYP nanoparticles, poly(CBA-ABOL) polyplexes	Hummers’ method (GO), co-precipitation (Fe_3_O_4_), polymer modification (oxidation + amination), electrostatic self-assembly	Positive surface charge, DNA condensation capacity, high surface area, multifunctionality (magnetic/photothermal)	Efficient nucleic acid complexation via electrostatic interactions; modular composition; potential external control (magnetic/NIR)	Cytotoxicity associated with cationic polymers (PEI); batch variability (GO synthesis); limited targeting specificity	[68,190,191]
Topographical and mechanotransduction platforms	Nanogrooved PUA/PDMS, microgrooved substrates, stiffness-tuned hydrogels	UV-assisted capillary lithography, soft lithography (PDMS), photolithography (SU-8), hydrogel stiffness tuning	Defined micro/nanotopography, tunable stiffness, surface anisotropy	Precise control of cell shape and cytoskeletal organization; reproducible patterning; modulation of mechanotransduction pathways	Limited biochemical functionality unless coated; fabrication may require specialized equipment; mostly 2D systems	[180,181,182,183,184]
3D supportive hydrogels and scaffolds	HA/gelatin hydrogels, chitosan-based hydrogels, pullulan nanogels, PLGA membranes	Photocrosslinking (PEGDA), mechanochemical grafting, chemical gelation, lyophilization, VIPS process (PLGA)	High water content, ECM-like structure, porosity, tunable crosslinking density	Biomimetic environment supports cell viability; tunable porosity and mechanics; compatibility with 3D culture and microfluidics	Limited mechanical strength (natural polymers); potential batch variability; degradation kinetics can be difficult to control	[185,186,187,188,189]
Controlled-release biomaterials	PLLA electrospun scaffolds + PEI-miRNA, IKVAV peptide hydrogels	Electrospinning, physical adsorption, self-assembly (pH-triggered), peptide design	Nanofibrous architecture, tunable mesh size, diffusion-controlled release, shear-thinning behavior	Spatially localized delivery; tunable release kinetics via structure and crosslinking; minimally invasive delivery (injectable hydrogels)	Limited control over long-term release profiles; potential burst release; dependence on diffusion rather than active targeting	[192,194]
Targeted nanocarrier systems (advanced delivery)	AuNP-based antibody-targeted nanocomplexes, biomimetic MSN-liposomes	Turkevich synthesis (AuNPs), thiol-gold chemistry, PEGylation, lipid coating, membrane protein functionalization	Precise size control, high surface functionalization, modular ligand conjugation, colloidal stability	High ligand density enables targeting; independent tuning of surface properties; robust structure under biological conditions	Non-biodegradable cores (AuNPs); complex multi-step fabrication; potential long-term accumulation and safety concerns	[193,195]

**Table 3:** Conceptual framework guiding biomaterial-assisted direct cellular reprogramming and functional integration.

Barrier	Underlying Biological Problem	Biomaterial-Based Strategy
Induction Phase: Low efficiency of conversion Poor stability of reprogramming factors Immunoreactivity	Epigenetic resistance, metabolic stress, apoptosis, inefficient MET Rapid miRNA/protein clearance and degradation Viral vector clearance, inflammatory signaling	Durable release of reprogramming factors; stiffness modulation; pro-MET biofunctionalization; antioxidant incorporation Functional stabilization, sustained and local delivery of reprogramming factors (hydrogels, nanoparticles, films and other scaffold-based reservoirs) Local confinement of vectors, delivery of immunomodulatory molecules
Incomplete lineage specification and cellular heterogeneity during conversion	Partial epigenetic remodeling, metabolic vulnerability	Topographical cues, stiffness matching the target tissue, ECM-inspired functionalization
Incomplete fate stabilization	Epigenetic drift, incomplete transgene silencing	Sustained microenvironmental signaling, hybrid culture systems integrating functional supportive cells or engineered tissue-like niches
Poor integration with the host	Mechanical mismatch, lack of vascularization, weak functional coupling (electrical/metabolic)	Tunable porosity, control of biomaterial degradation, functionalization with angiogenic and chemoattractant molecules, endothelial progenitors, conductive scaffolds (excitable tissues)

## Data Availability

All data discussed in this review are derived from previously published studies, which are cited accordingly. No new data were generated.
